# Lymphatic Endothelial Cells in Health and Disease

**DOI:** 10.1002/mco2.70891

**Published:** 2026-07-27

**Authors:** Du Tang, Wang Lin, Hengyang Liu, Jixin Zhong, Shiliang Li, Xiaoquan Rao

**Affiliations:** ^1^ Division of Cardiology Department of Internal Medicine Tongji Hospital, Tongji Medical College, Huazhong University of Science and Technology Wuhan Hubei China; ^2^ Department of Cardiology Renmin Hospital of Wuhan University Wuhan Hubei China; ^3^ Division of Rheumatology Fujian Medical University Union Hospital Fuzhou China; ^4^ Department of Internal Medicine Tongji Hospital, Tongji Medical College, Huazhong University of Science and Technology Wuhan China; ^5^ Division of Cardiothoracic and Vascular Surgery Tongji Hospital, Tongji Medical College, Huazhong University of Science and Technology Wuhan Hubei China

**Keywords:** heterogeneity, immune regulation, lymphangiogenesis, lymphatic endothelial cells, lymphatic remodeling, tissue homeostasis

## Abstract

Lymphatic endothelial cells (LECs) line the lymphatic vasculature and support interstitial fluid drainage, lipid transport, and immune‐cell trafficking. Beyond these classical functions, recent studies now recognize LECs as heterogeneous, spatially organized endothelial regulators that maintain lymphatic identity while adopting tissue‐, segment‐, and disease‐associated states. Advances in lineage tracing, multiomics, functional imaging, and perturbation studies now link LEC heterogeneity to drainage control, immune surveillance, antigen handling, metabolic homeostasis, and tissue repair. Despite these advances, a unified framework explaining how developmental origin, tissue niche, and context‐dependent state transitions collectively shape LEC function across physiology and disease remains lacking. In this review, we summarize the developmental origins, identity‐maintenance mechanisms, and anatomical deployment of LEC states across the lymphatic network. We then discuss how specialized LEC states coordinate lymphatic transport, immune surveillance, and peripheral tolerance under homeostasis. We further examine how context‐dependent LEC reprogramming contributes to lymphatic disorders, inflammation and autoimmunity, cancer, cardiometabolic disease, and central nervous system dysfunction. Finally, we highlight challenges and opportunities for translational LEC‐targeted therapy. Together, this state‐centered framework reframes LECs as actionable regulators of tissue homeostasis and disease progression, providing a conceptual foundation for precision lymphatic medicine.

## Graphical abstract

Lymphatic endothelial cells (LECs) are dynamic regulators of tissue homeostasis and disease remodeling. In the healthy state, LECs coordinate interstitial fluid drainage, intestinal lipid transport, and immune trafficking and tolerance. Across disease settings, however, these same programs become maladaptively reprogrammed, contributing to barrier dysfunction, chronic inflammation, tumor‐associated lymphatic remodeling, and altered antigen handling. This conceptual framework highlights LEC state transitions as a central axis linking lymphatic function to pathogenesis and therapeutic opportunity.

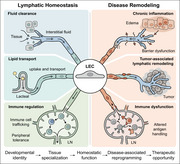

## 1 Introduction

Lymphatic endothelial cells (LECs) constitute the cellular interface of the lymphatic vasculature. Historically, their biological importance was interpreted mainly through the most visible and classical functions of the lymphatic system, including interstitial fluid uptake, tissue fluid return, and lymphatic drainage [1, 2, 3, 4]. Within this framework, the biological significance of LECs was naturally inferred from the structural presence of lymphatic vessels and their contribution to fluid balance. This view established an essential foundation for lymphatic biology, but it is now too limited to explain the broader regulatory roles of LECs in tissue organization, organ homeostasis, and disease progression [5, 6, 7, 8].

Recent advances have substantially expanded this traditional view. Developmental lineage‐tracing studies have refined the origins and diversification of LECs [9, 10, 11, 12], and single‐cell and spatial multiomic analyses have uncovered pronounced heterogeneity among LECs, identifying tissue‐specific subsets with distinct transcriptional programs, including immune‐interacting, lipid‐handling, and barrier‐regulating phenotypes [13, 14, 15]. Functional studies further demonstrate that LECs actively participate in antigen presentation, immune tolerance, and microenvironmental adaptation, rather than serving solely as passive conduits [5, 6, 16, 17].

These findings collectively shift the focus of the field from a structure‐centric view of lymphatic vessels toward a state‐centered understanding of LEC biology. A central implication of this shift is that lymphatic structure is not synonymous with lymphatic function. Structural features such as vessel density, dilation, or remodeling remain informative, but they are often insufficient to predict functional outcomes [18, 19]. Similar morphological changes may reflect fundamentally different biological states depending on tissue context, disease stage, and the underlying state of LECs [6, 8, 18]. For example, lymphatic expansion may support immune resolution in some settings while facilitating tumor dissemination or chronic inflammation in others [20, 21]. Therefore, the key question is no longer simply whether lymphatic vessels form or regress, but how LEC states are established, maintained, and dynamically reprogrammed, and how these transitions shape core physiological processes, including fluid clearance, immune coordination, lipid metabolism, and tissue repair.

With this shift in view, this review is organized around the relationship between LEC lineage, molecular identity, cellular state, function, and disease remodeling. We first examine how LEC identity is established from diverse developmental origins, stabilized by core molecular programs, and diversified into anatomically deployed states within the lymphatic network. We then discuss the principal physiological functions of LECs under homeostasis, emphasizing how these functions are deployed across tissues and sustained by distinct cellular states. We next consider how LEC programs are reconfigured across major disease settings, with particular attention to the transition from adaptive remodeling to functional decompensation and pathological progression. Finally, we highlight emerging therapeutic strategies and imaging‐based translational opportunities that arise from a more state‐aware view of LEC biology, underscoring the potential of these cells as precision therapeutic targets.

## 2 Development and Characterization of Lymphatic Endothelium

**FIGURE 1:**
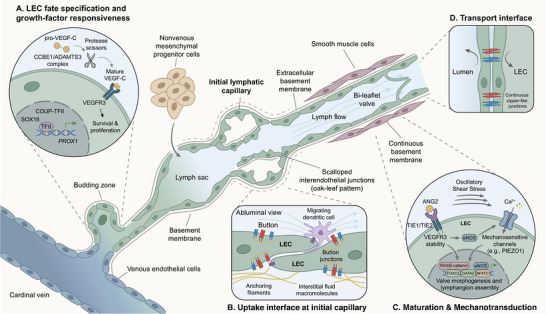
Ontogeny, maturation, and transport interfaces of lymphatic endothelial cells. (A) Lymphatic endothelial cell (LEC) specification from venous endothelium is driven by SOX18, COUP‐TFII, PROX1, and VEGFC–VEGFR3 signaling. (B) Initial lymphatic capillaries form an uptake interface characterized by a discontinuous basement membrane, anchoring filaments, and button‐like interendothelial junctions. (C) Collecting lymphatic vessels acquire smooth muscle coverage, a continuous basement membrane, bileaflet valves, and flow‐responsive signaling involving ANG2, TIE1/TIE2, PIEZO1, eNOS, FOXC2, GATA2, and NFATC1. (D) Mature collecting lymphatic vessels display continuous zipper‐like junctions that support barrier integrity, low‐leakage conveyance, and directional lymph flow. *Abbreviations*: ADAMTS3, a disintegrin and metalloproteinase with thrombospondin motifs 3; ANG2, angiopoietin 2; CCBE1, collagen and calcium‐binding EGF domains 1; COUP‐TFII, chicken ovalbumin upstream promoter‐transcription factor II; eNOS, endothelial nitric oxide synthase; LEC, lymphatic endothelial cell; NFATC1, nuclear factor of activated T cells 1; PIEZO1, piezo type mechanosensitive ion channel component 1; TIE1, tyrosine kinase with immunoglobulin‐like and EGF‐like domains 1; TIE2, tyrosine kinase with immunoglobulin‐like and EGF‐like domains 2; VEGFC, vascular endothelial growth factor C; VEGFR3, vascular endothelial growth factor receptor 3.

Lymphatic endothelium is traditionally defined by its embryonic ontogeny, canonical markers, and characteristic vessel morphology. While these features provide a foundational framework, they do not fully capture the dynamic processes through which a functional, hierarchically organized, and regionally specialized lymphatic system is established and maintained. This section synthesizes current understanding across three interconnected themes: the molecular specification of LEC identity, the stabilization of this identity amidst lineage diversification, and the deployment of distinct LEC states within a specialized transport network (Figure 1).

### 2.1 Ontogeny and Morphogenesis of the Lymphatic Vasculature

#### 2.1.1 Lineage Specification and Identity Maintenance

The classical paradigm of lymphatic development proposes that LECs arise predominantly from a specialized subset of venous endothelial cells in the cardinal vein, which bud to form the primitive lymph sacs and subsequently generate the early lymphatic vasculature [22, 23, 24, 25, 26, 27]. Compelling evidence for this model stems from the characterization of Prospero homeobox 1 (PROX1), which selectively marks the initial venous endothelial cells committed to lymphatic fate and is required to initiate the lymphatic developmental program [22, 27, 28]. During this early specification step, SRY‐box transcription factor 18 (SOX18) cooperates with nuclear receptor subfamily 2 group F member 2 (NR2F2) to induce PROX1 expression and thereby initiate LEC differentiation [25, 29] (Figure 1A). Once induced, PROX1 functions as a molecular anchor that stabilizes the lymphatic program by sustaining a suite of canonical markers, including Fms‐related receptor tyrosine kinase 4 (FLT4)/vascular endothelial growth factor receptor 3 (VEGFR3), neuropilin 2 (NRP2), podoplanin (PDPN), and lymphatic vessel endothelial hyaluronan receptor 1 (LYVE1), thereby linking initial fate specification to long‐term lymphatic identity maintenance [29, 30, 31, 32, 33, 34, 35].

#### 2.1.2 Chimeric Origins of Lymphatic Beds

Although venous origin remains the dominant framework, it is no longer sufficient to account for the full diversity of lymphatic beds. Recent lineage‐tracing studies have refined this view by uncovering the chimeric nature of the lymphatic system, demonstrating that in organs such as the skin, mesentery, and heart, LECs can additionally arise from nonvenous sources, including mesenchymal or hematopoietic endothelial‐associated populations, as well as from ISL LIM homeobox 1^+^ second heart field cells [9, 10, 11, 36, 37]. Evidence has further shown that a subset of early LECs can be specified directly from ETV2^+^PROX1^+^ mesenchymal progenitors of paraxial mesodermal origin [12]. The emerging consensus, therefore, is that lymphatic development follows an organ‐specific mosaic model: while predominantly venous in origin, it is supplemented by diverse progenitor sources to meet the unique physiological demands of different tissue niches [12, 27].

#### 2.1.3 Network Expansion, Separation, and Specialization

Once LEC fate is specified, the primitive network expands under the control of the vascular endothelial growth factor C (VEGFC)–VEGFR3 axis [23, 35]. This process requires proteolytic maturation of VEGFC by collagen and calcium‐binding EGF domain‐containing protein 1 (CCBE1) and A disintegrin and metalloproteinase with thrombospondin motifs 3 (ADAMTS3) [38]. A critical developmental checkpoint is the physical and functional separation of the lymphatic system from the venous system, a process mediated by platelet–LEC interactions and PDPN signaling [39, 40, 41, 42, 43]. Development then shifts from expansion to hierarchical remodeling, in which lymph flow, growth factor signaling, and flow‐responsive transcriptional programs drive collecting‐vessel maturation and intraluminal valve initiation [44, 45, 46, 47, 48, 49, 50] (Figure 1C). Importantly, full collecting‐vessel function is acquired only after later maturation of the antireflux valve apparatus [51]. Spatial patterning adds a further layer to this process. NRP2 enhances VEGFC‐dependent sprouting behavior [32, 52], whereas guidance signals such as semaphorin 3F (SEMA3F) and semaphorin 3G (SEMA3G) restrict excessive branching and help define lymphatic pathfinding boundaries [53, 54]. More recent evidence further suggests that C–X–C motif chemokine ligand 12 (CXCL12)–C–X–C chemokine receptor type 4 (CXCR4) signaling contributes to this patterning logic by regulating lymphatic sprouting, migration, and valve‐associated development in a context‐dependent manner [55]. The culmination of this program is the emergence of organ‐specific transport interfaces, including intestinal lacteals specialized for lipid transport, lymphoid LECs optimized for immune‐cell trafficking, and dermal lymphatics tailored for interstitial clearance [15, 56, 57, 58]. Taken together, these developmental events do not merely generate lymphatic vessels, but progressively convert lineage specification into a hierarchically patterned, organ‐adapted transport system.

### 2.2 Molecular Identity and Organized Heterogeneity of LECs

While embryonic ontogeny defines the developmental origins of LECs, it does not, in isolation, account for how lymphatic functions are distributed across tissues, vessel segments, and physiological states. More fundamentally, LEC identity is not a static collection of markers but a stabilized molecular program that maintains core lymphatic features while allowing for the specialization required in different tissue environments. The central conceptual challenge, therefore, shifts from the mere identification of LECs to understanding how a foundational lymphatic blueprint is maintained, spatially diversified, and selectively reconfigured to fulfill organ‐ and segment‐specific demands.

#### 2.2.1 Core LEC Identity Program and Canonical Markers

PROX1 serves as the principal regulator of lymphatic endothelial specification and lifelong identity maintenance. It drives the transition of venous endothelial cells toward the lymphatic lineage and must remain active to preserve the differentiated LEC state, since loss of PROX1 is sufficient to reprogram LECs toward a blood endothelial phenotype [22, 28, 59, 60, 61, 62]. However, maintenance of lymphatic identity does not depend on PROX1 alone. Recent work has shown that the cooperative activity of ETS transcription factor ERG (ERG) and Fli‐1 proto‐oncogene, ETS transcription factor (FLI1), is required to preserve lymphatic integrity, sustain transcriptionally distinct LEC populations, and maintain core lymphatic gene programs in adult vessels [63]. The PROX1–VEGFR3 feedback loop further ties transcriptional identity to receptor‐level stabilization of lymphatic fate, indicating that identity maintenance is reinforced by reciprocal regulatory interactions rather than by a single master factor alone [35]. This mutual reinforcement renders the lymphatic program stable under homeostatic conditions yet sensitive to disease‐associated cues that perturb this regulatory axis.

From the standpoint of molecular identification, VEGFR3/FLT4, LYVE1, and PDPN remain the most widely used canonical markers for distinguishing LECs from blood endothelial cells (BECs) [64, 65, 66]. NRP2 is likewise frequently incorporated into this classical lymphatic marker framework, particularly in developmental and sprouting settings [32]. Yet these molecules are best understood as the surface manifestation of a coordinated lymphatic gene program rather than as an arbitrary collection of positive labels [67, 68, 69, 70].

#### 2.2.2 Single‐Cell and Spatio‐Omics Reveal Internal Heterogeneity in LECs

A shared core lymphatic program does not mean that all LECs exist in the same state. Rather, single‐cell and spatial studies now show that LEC heterogeneity is organized by anatomical location, vessel segment, and organ context [5, 13, 71]. The clearest evidence comes from lymph nodes, where both mouse and human analyses identify distinct LEC populations in the ceiling and floor of the subcapsular sinus, medullary sinuses, and cortical lymphatic niches. Crucially, these studies do not simply subdivide LECs into clusters; they link spatially positioned subsets to local functions, including immune‐cell trafficking, scavenging, and antigen archiving [5, 14, 15]. In mice, cortical sinus LECs expressing annexin A2 (*Anxa2*), pentraxin 3 (*Ptx3*), potassium inwardly rectifying channel subfamily J member 8 (*Kcnj8*), and inter‐alpha‐trypsin inhibitor heavy chain 5 (*Itih5*) are associated with rapid lymphocyte egress [14, 72], whereas in humans, *CD209*‐expressing medullary LECs bind neutrophils through Lewis X, indicating that at least some subset‐associated markers correspond to defined leukocyte‐handling functions rather than descriptive transcriptional labels [15].

This position–function relationship becomes even clearer when regional signaling programs are taken into account. Atypical chemokine receptor 4 (ACKR4), enriched in defined lymphatic territories, shapes local C–C chemokine receptor 7 (CCR7)‐ligand gradients through chemokine scavenging: in lymph node subcapsular sinus ceiling LECs it stabilizes interfollicular C–C motif chemokine ligand 21 (CCL21) gradients that direct lymph‐borne CCR7^+^ antigen‐presenting cells into the parenchyma, whereas in peripheral lymphatic tissues and inflamed collectors it regulates CCL19/CCL21 availability and thereby supports dendritic‐cell (DC) emigration and T‐cell transport [56, 73, 74, 75]. More recent work combining single‐cell sequencing with spatial transcriptomics showed that ceiling LECs (cLECs) and floor LECs (fLECs) occupy defined microanatomical sites with particularly strong antigen‐archiving capacity and characteristic immune‐interaction programs [5, 76, 77]. Spatial information therefore does more than annotate anatomy; it reveals that LEC state is physiologically organized by position.

Likewise, in the developing heart, single‐nuclei multiomic analysis identified cardiac LEC populations associated with epicardial and coronary artery niches [71]. In adult skin, 3D imaging combined with single‐cell transcriptomics revealed a hierarchical capillary‐to‐collecting organization together with two distinct valve LEC clusters [13]. Adult Forkhead box C2 (FOXC2)‐dependent specialization further indicates that differentiated LEC states are actively maintained rather than passively retained after development [78]. Taken together, these findings indicate that LEC heterogeneity is functionally organized rather than merely descriptively clustered: distinct LEC states are positioned across organs and transport segments to support specialized tasks, and these same states can be selectively reconfigured in disease [6, 79] (Table 1).

**TABLE 1:** Representative LEC populations across anatomical niches, with representative markers/features and functions.

LEC population and niche	Representative markers and features	Principal functions	Biological significance	References
Foundational/shared LEC state across lymphatic beds	*PROX1–FLT4/VEGFR3* core identity axis; *PDPN* and *LYVE1* as canonical markers; *ERG/FLI1*‐dependent identity reinforcement	Establishes and stabilizes lymphatic endothelial identity	Provides a common basis for later specialization across lymphatic beds	[22, 31, 35, 63, 65]
Subcapsular sinus cLECs and fLECs, lymph node ceiling and floor	Mouse: *Ackr4*, *Anxa2*, *Cd36* (cLEC); *Lyve1*, *Madcam1*, *Itga2b* (fLEC); ceiling/floor specialization	Chemokine shaping, immune‐cell entry, antigen handling	Microanatomical immune specialization	[14, 73, 75, 80]
Cortical and medullary sinus LECs, lymph node cortical and medullary regions	Mouse: *Ptx3*‐associated cortical egress program; *Il33*/*Mrc1*/*Marco*‐associated medullary; Human: *CD209*‐enriched medullary subset	Lymphocyte egress, scavenging and leukocyte interaction	Compartment‐specific nodal specialization	[14, 15, 72]
Antigen‐archiving sinus LEC states, lymph node sinus niches	Antigen‐archiving‐associated endocytic/caveolar program; *CAV1*‐associated archiving	Antigen retention and stromal support of recall responses	Durable immune‐memory support	[5, 76, 77, 81]
Initial capillary LECs, peripheral lymphatic capillary beds	*LYVE1*, *FLT4*/*VEGFR3*, *PDPN*; button‐like uptake interface	Fluid, macromolecule, and immune‐cell uptake	Tissue‐entry module	[82, 83, 84, 85, 86]
Collecting and valve‐associated LECs, downstream collectors and valve regions	*FOXC2*, *GATA2*; lymphangion organization and flow‐responsive specialization	One‐way transport and antireflux control	Downstream transport module	[13, 48, 50, 78]
Lacteal LECs, intestinal villi	*LYVE1*, *FLT4*/*VEGFR3*, *PROX1*; lipid‐handling and intestinal segment‐associated specialization	Dietary lipid uptake and transport	Organ‐adapted metabolic specialization	[57, 78, 87, 88, 89]
Cardiac LEC populations, epicardial and coronary‐associated niches	Human fetal heart: *PROX1*, *RELN*; injury‐associated state remodeling	Fluid clearance, inflammatory resolution, tissue repair	Injury‐driven state reorganization	[71, 90, 91, 92]
Meningeal LECs, meningeal lymphatic vessels	*PROX1*, *LYVE1*, *PDPN*, *FLT4*/*VEGFR3*; CNS‐associated drainage specialization	CNS fluid and macromolecule clearance	CNS clearance–immune coupling	[93, 94, 95, 96]
Disease‐remodeled LEC states, inflamed or tumor‐associated beds	Inflammation‐ or tumor‐induced immune‐regulatory reprogramming; checkpoint‐ and Treg‐associated tumor LEC programs	Immune modulation and transport remodeling	Selective pathological reprogramming	[6, 79, 97, 98, 99]

*Abbreviations*: ACKR4, atypical chemokine receptor 4; cLEC, ceiling lymphatic endothelial cell; CNS, central nervous system; DC, dendritic cell; fLEC, floor lymphatic endothelial cell; FLT4, fms‐related receptor tyrosine kinase 4; FOXC2, forkhead box C2; GATA2, GATA binding protein 2; LEC, lymphatic endothelial cell; LYVE1, lymphatic vessel endothelial hyaluronan receptor 1; PDPN, podoplanin; PROX1, prospero homeobox 1; RELN, reelin; VEGFR3, vascular endothelial growth factor receptor 3.

#### 2.2.3 Metabolic and Epigenetic Maintenance of LEC Identity and State Responsiveness

LEC identity is often discussed in relation to lineage‐defining transcription factors, but this view remains incomplete. Rather than being maintained by PROX1 alone or lost through simple transcriptional drift, lymphatic identity is better understood as an actively sustained state supported by metabolic and chromatin regulation. Metabolic pathways do more than provide energy, and epigenetic mechanisms do more than lock in fate; together, they preserve the accessibility, responsiveness, and continuity of the core lymphatic program, as supported by studies linking fatty acid β‐oxidation, mitochondrial respiration, lipophagy, cholesterol‐dependent control of VEGFR3 signaling, chromatin accessibility at PROX1 regulatory regions, and stress‐responsive chromatin remodeling to maintenance of lymphatic fate and adaptive state control [60, 100, 101, 102, 103, 104, 105, 106, 107].

One major dimension of this regulation is metabolic. A central mechanism is the coupling of cellular metabolism to the transcriptional maintenance of lymphatic fate. Fatty acid β‐oxidation supplies acetyl‐CoA for histone acetylation, thereby enabling PROX1 together with p300/EP300 to activate lymphatic regulatory elements while suppressing the blood endothelial program [100]. This transcriptional support, however, depends on broader metabolic fitness: mitochondrial respiration sustains the PROX1–VEGFR3 feedback loop, and its disruption impairs both fate specification and identity maintenance [60], while lipophagy preserves fatty acid oxidation and acetyl‐CoA availability, thereby maintaining PROX1 target gene expression and lymphangiogenic responsiveness [101]. Beyond supporting transcriptional maintenance, metabolic control also extends to receptor‐level signaling competence. Cholesterol handling, in this sense, is instructive rather than merely permissive: apolipoprotein A‐I binding protein‐mediated cholesterol efflux promotes LEC fate specification and lymphangiogenesis by relieving caveolae‐mediated inhibition of VEGFR3 signaling, whereas LEC‐specific disruption of the sphingosine 1‐phosphate (S1P)/sterol regulatory element‐binding protein 2 (SREBP2)‐dependent cholesterol biosynthetic program reduces VEGFR3 expression and results in defective lymphatic development and postnatal lymphatic dysfunction [102, 103]. Together, these findings indicate that metabolic regulation supports LEC identity at multiple levels, from chromatin‐linked transcriptional maintenance to preservation of VEGFR3‐dependent signaling competence.

A second layer resides in chromatin organization and regulatory architecture. This is not merely a downstream consequence of lineage commitment, but a central mechanism through which the lymphatic program is maintained and refined. Zinc finger MIZ‐type containing 1 (ZMIZ1), for example, is increasingly recognized as a regulator of LEC gene expression and function, and loss of ZMIZ1 reduces PROX1 expression, impairs LEC migration and proliferation, decreases chromatin accessibility at PROX1 regulatory regions, and lowers mesenteric lymphatic valve density in vivo [104]. Consistent with this, developmental cis‐regulatory analysis in zebrafish showed that *Prox1a* expression is controlled by multiple enhancers with bed‐specific activity rather than by a single pan‐lymphatic element; notably, a valve‐associated enhancer was required for proper valve leaflet morphology and function, indicating that upstream regulation of the PROX1/PROX1a axis is spatially modular and functionally consequential [105]. What is preserved in LECs, therefore, is not simply expression of a canonical marker set, but a regulatory architecture that remains accessible, spatially tuned, and responsive to context.

This regulatory logic extends to the epigenomic level. Primary human LECs and BECs display distinct DNA methylation and histone modification landscapes, indicating that lineage stability is embedded in a lineage‐specific chromatin state rather than in transcriptional output alone. Yet this state is not absolutely sealed, because perturbation of DNA methylation and histone methylation preferentially induces blood endothelial markers in LECs, suggesting that endothelial lineage specificity is epigenetically reinforced while retaining limited asymmetric plasticity [108, 109]. Epigenetic regulation in LECs, therefore, functions less as a rigid lock than as a stabilizing system that preserves lineage continuity while permitting constrained responsiveness to perturbation. Consistent with this view, a recent multiomics study showed that blood and LECs maintain distinct metabolic programs across proliferative and quiescent states, supporting the idea that endothelial quiescence is actively maintained rather than passively assumed [110].

Taken together, these findings indicate that LECs are defined not simply by the presence of PROX1 and other canonical markers, but by a lymphatic identity program that is continuously sustained, constrained, and selectively reshaped through metabolic and epigenetic regulation. In this sense, lineage continuity is preserved not despite adaptability, but through it. LEC identity is not a residual developmental imprint, but an actively maintained regulatory state whose stability depends on continuous metabolic and chromatin‐level support.

### 2.3 Structural Deployment of LEC States Within the Lymphatic Network

The mature lymphatic vasculature is often described as an anatomical division between initial capillaries and collecting vessels. Although this distinction is descriptively useful, it does not fully explain how lymph transport is organized once the network is established. Functionally, what matters is not merely the presence of two vessel classes, but the spatial deployment of distinct LEC states along a transport axis. The mature lymphatic network is therefore better understood as a coordinated transport continuum built on spatially specialized LEC states rather than as two static anatomical classes arranged in series.

At the tissue boundary, initial lymphatic capillaries are specialized for uptake via discontinuous button‐like junctions that create primary entry sites for fluid and immune cells [84, 85, 111], anchoring filaments that tether the endothelium to the surrounding matrix [2, 112, 113, 114], and minimal mural coverage characteristic of capillary lymphatics rather than collectors [115] (Figure 1B). Importantly, this entry interface is not constitutively open, but is dynamically regulated. In lacteals, chylomicron‐derived lipids promote junction opening through Rho‐associated coiled‐coil‐containing protein kinase (ROCK)‐dependent contraction of junction‐anchored stress fibers, whereas vascular endothelial growth factor A (VEGFA) counterbalances this response through VEGFR2/VEGFR3–PI3K–AKT–RAC1 signaling, thereby establishing a regulatable barrier for lipid entry [89]. Endothelial O‐GlcNAcylation provides a related regulatory mechanism; its loss induces a button‐to‐zipper transition, reduces VEGFR3 expression, and impairs dietary fat absorption [116]. In dermal initial lymphatics, elevated VEGFA similarly drives VE‐cadherin zippering and fragmentation through Src‐dependent junctional remodeling, changes that are associated with tumor cell entry into lymphatic vessels and dissemination to sentinel lymph nodes [117]. Together, these findings indicate that entry‐site transport depends on active junctional remodeling rather than on a permanently open structure.

By contrast, downstream collecting segments contain LEC states specialized for containment and low‐leakage conveyance. Compared with initial capillaries, these segments display tighter wall organization, more continuous junctions, a more complete basement membrane, and support from lymphatic muscle cells, features better suited to low‐leakage transport over distance [47, 85, 118] (Figure 1D). This logic is especially evident in propulsion, where valve‐associated endothelial organization, together with lymphatic muscle, supports forward lymph movement. Recent physiological and cellular studies indicate that lymphatic muscle contributes intrinsic pacemaking, whereas valve function is essential for maintaining net forward transport [119, 120, 121, 122]. The key distinction, therefore, is not simply between more permeable and less permeable vessels, but between endothelial states specialized for entry, containment, and coordinated downstream propulsion.

This hierarchy, however, is not fully captured by a simple transition from entry to propulsion. Between uptake and downstream pumping lies a specialized intermediate zone that is not fully captured by the conventional capillary‐versus‐collector dichotomy. Earlier work identified specialized lymphatic endothelial features in skin precollectors [123]. More recent 3D imaging combined with single‐cell transcriptomics further showed that the capillary‐to‐precollector boundary contains multiple valves and two transcriptionally distinct valve LEC clusters. These findings support the view that this region represents a specialized transition domain within the transport axis rather than a purely mechanical checkpoint [13].

This spatial organization of distinct LEC states extends from local vessel hierarchy to organ‐scale routing. In the intestine, regionally distinct lacteal LEC states help determine the efficiency of dietary lipid uptake and transport, indicating that mature lymphatic performance depends on more than the mere presence of lacteals as a structural feature [57]. In the central nervous system (CNS), drainage is similarly organized through nonequivalent outflow routes: deep cervical outflow can occur spontaneously in relation to intracranial pressure, whereas superficial cervical drainage depends more strongly on lymphatic pumping, and human tissue mapping identifies a compartmentalized ventral dural outflow zone around the middle meningeal artery [124, 125]. These observations extend the concept of LEC heterogeneity beyond local vessel segments and support the view that tissue‐specific lymphatic function also depends on organ‐adapted routing programs.

Overall, the mature lymphatic network is best understood as an integrated transport system comprising four coupled modules: regulated uptake at the entry interface, low‐leakage downstream conveyance, valve‐ and muscle‐dependent propulsion, and organ‐adapted routing. This framework moves beyond a static two‐class anatomical model and emphasizes that effective lymph transport depends on the coordinated deployment of distinct LEC states across the network.

## 3 Functional Physiology of LECs in Homeostasis

**FIGURE 2:**
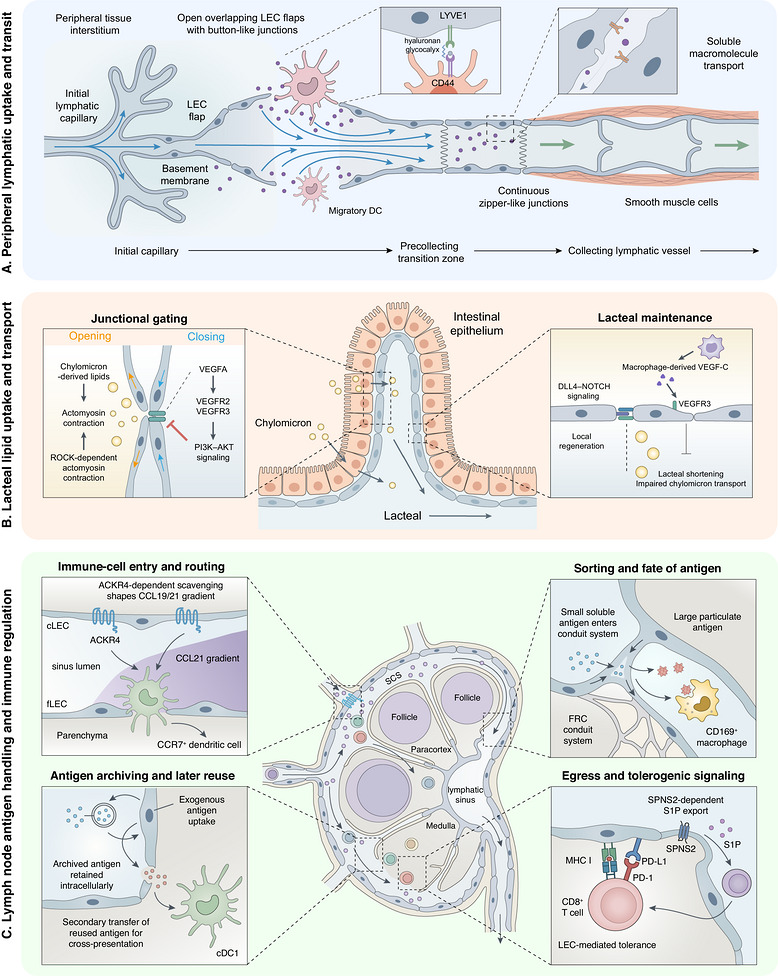
State‐dependent lymphatic endothelial cell functions across fluid, lipid, and immune interfaces. (A) In peripheral tissues, LECs support fluid and macromolecule clearance through a regulated uptake interface, LYVE1–CD44‐dependent recognition, and downstream collector‐mediated transport. (B) In the intestine, lacteal LECs mediate lipid uptake and chylomicron transport through junctional gating, local contractile activity, and niche‐derived maintenance signals, including VEGFC–VEGFR3 and DLL4–NOTCH pathways. (C) In lymph nodes, distinct LEC subsets regulate immune‐cell migration, antigen access and archiving, lymphocyte egress, and tolerance‐related signaling through chemokine shaping, S1P–SPNS2‐dependent egress cues, antigen presentation, and PD‐L1–PD‐1 signaling. ACKR4, atypical chemokine receptor 4; CCL19/21, C–C motif chemokine ligand 19/21; CCR7, C–C motif chemokine receptor 7; CD44, cluster of differentiation 44; CD169, cluster of differentiation 169; cDC1, type 1 conventional dendritic cell; cLEC, ceiling lymphatic endothelial cell; DC, dendritic cell; DLL4, delta‐like ligand 4; FRC, fibroblastic reticular cell; fLEC, floor lymphatic endothelial cell; LEC, lymphatic endothelial cell; LYVE1, lymphatic vessel endothelial hyaluronan receptor 1; MHC I, major histocompatibility complex Class I; PD‐1, programmed cell death protein 1; PD‐L1, programmed death‐ligand 1; S1P, sphingosine‐1‐phosphate; SCS, subcapsular sinus; SMCs, smooth muscle cells; SPNS2, spinster homolog 2; VEGFA, vascular endothelial growth factor A; VEGFC, vascular endothelial growth factor C; VEGFR2, vascular endothelial growth factor receptor 2; VEGFR3, vascular endothelial growth factor receptor 3.

Specialized LEC states convert the lymphatic network from an anatomical conduit into a homeostatic transport system. Mature lymphatic function depends on the coordinated deployment of LEC programs across vessel segments and tissue niches, supporting fluid and macromolecule clearance, downstream containment and propulsion, dietary lipid transport, immune‐cell trafficking, antigen handling, and peripheral tolerance (Figure 2).

### 3.1 Interstitial Fluid and Macromolecule Clearance

Among the core physiological functions of the lymphatic vasculature, the return of interstitial fluid and macromolecules is fundamental [7]. However, this function is neither executed uniformly across organs nor explained simply by the presence of lymphatic vessels. Recent work instead suggests that clearance is shaped by the tissue‐specific deployment of distinct LEC states, with local structural and molecular features determining how efficiently fluid and soluble cargo are taken up, conveyed, and routed [126, 127]. Under homeostatic conditions, these tasks are partitioned across the lymphatic tree: initial capillary LECs form the uptake interface for interstitial fluid and soluble cargo, collector‐associated LEC states support downstream containment and transport through valve‐delimited lymphangions, and organ‐specific drainage architectures guide tissue outflow [85, 119, 121, 126, 127] (Figure 2A). Interstitial clearance is therefore best understood not as a uniform vessel property, but as a state‐dependent network function that maintains tissue fluid balance, colloid osmotic homeostasis, and systemic fluid equilibrium [7, 128].

The efficiency of this process depends on coordinated performance across these compartments rather than on vessel presence alone. Lymphatic dysfunction may therefore emerge before overt vessel loss, initially as impaired transport. This is particularly evident at the entry interface, where capillary LEC junctions can reversibly shift between button and zipper configurations during postnatal maturation and inflammation, accompanied by changes in endothelial morphology and vessel diameter [111]. In mouse airway mucosa, button junctions are progressively established after birth, whereas allergic inflammation drives a return toward zippering [111]. This junctional state is actively regulated rather than passively maintained: VEGFR3 signaling promotes button formation, whereas pathological VEGFA signaling drives zippering of initial lymphatics. Consistently, loss of VEGFR3 signaling disrupts button junctions and impairs uptake of interstitial molecules across multiple tissues [84, 86, 117]. Pathological zippering likewise reduces fluid transport in infection and other disease settings [85, 86, 111, 129]. These findings indicate that absorptive capillary LEC states remain plastic after formation and that clearance can fail early at the level of capillary uptake.

Macromolecule handling at this interface is also not determined by junctional openness alone. LYVE1, a classical lymphatic surface receptor, mediates hyaluronan‐dependent interactions at the luminal surface of initial lymphatics, and the cluster of differentiation 44 (CD44)–hyaluronan glycocalyx on migrating DCs cooperates with endothelial LYVE1 to regulate entry into lymphatic capillaries [30, 130]. Structural study has further defined an unusual LYVE1–hyaluronan binding mechanism that supports leukocyte docking and entry [131]. Thus, entry‐site LECs provide not only a permissive permeability interface but also a selective recognition interface that shapes how glycocalyx‐rich cellular and macromolecular cargo engage the vessel wall.

Entry, however, is only the first step in effective clearance. Once fluid and macromolecules enter the network, efficient transport depends on collector segments specialized for downstream containment and forward propulsion. Mature collecting vessels do not simply receive lymph passively; they support lymphangion‐based pumping, in which each valve‐delimited segment couples rhythmic wall contraction to unidirectional flow [132]. Recent work refined this framework by showing that lymphatic muscle cells act as the intrinsic pacemaker population that initiates spontaneous contractions in mouse collecting lymphatics [121]. Complementary physiological studies further showed that pressure conditions dominate contraction‐wave generation and propagation, whereas intraluminal valves improve net forward transport by limiting reverse movement under intrinsically driven flow [119, 120]. These findings support the view that collector function depends on coordinated coupling among collector LEC organization, valve competence, and active propulsion, rather than on vessel continuity alone.

Taken together, interstitial fluid and macromolecule clearance is not a single capillary function but an emergent network function of state‐dependent modules deployed across the lymphatic tree. Dysfunction may therefore arise from absorptive failure at the capillary inlet, loss of recognition capacity at the entry interface, impaired containment in collectors, defective valve coordination, or maladaptive rerouting of tissue outflow. Early lymphatic failure may therefore reflect disrupted functional coupling among LEC states across entry, conveyance, propulsion, and outflow compartments, often before overt structural regression. This state‐centered view better explains how edema, solute retention, and downstream tissue dysfunction can begin before frank network loss becomes apparent.

### 3.2 Lipid Absorption and Chylomicron Transport

Lacteals illustrate how specialized LEC states support dietary lipid uptake and delivery into the systemic circulation. This function is not organized uniformly along the intestine. Instead, recent work has revealed pronounced regional specialization, with duodenal lacteals displaying the most discontinuous junctional organization and contributing disproportionately to rapid dietary lipid uptake, whereas helminth‐induced junctional zippering markedly reduces absorptive efficiency [57]. More broadly, lacteal permeability itself has emerged as a determinant of intestinal lipid uptake [89]. These findings indicate that lipid absorption is shaped by intestinal segment identity and inflammatory state, rather than by vessel position alone (Figure 2B).

Positioned at the center of small‐intestinal villi, lacteals receive chylomicrons assembled by enterocytes and released into the lamina propria, after which these lipid‐rich particles are conveyed through mesenteric lymphatics and the thoracic duct into the bloodstream [133, 134]. Yet the efficiency of this pathway cannot be reduced to particle entry alone. Rather, it depends on coordinated coupling across the lacteal‐associated transport axis, including a permissive uptake interface, effective downstream drainage, and sufficient mechanical activity to sustain forward movement of absorbed cargo. Consistent with this view, lacteal junction opening is required for plasma lipid uptake, whereas VEGFR3‐ and myosin light chain 2 (MLC2)‐dependent contractile responses support lymph flow and triglyceride output [89, 135, 136]. Thus, chylomicron transport reflects functional coupling between absorptive and transport‐related LEC states from entry to efflux.

This coupling is also more actively regulated than earlier static models had implied. Intravital imaging showed that postprandial lipid drainage is closely linked to lacteal contractility, with surrounding smooth muscle fibers providing the structural basis for this rhythmic activity; when contraction is suppressed, lipid drainage falls and retained lipid accumulates within villi [134]. More recent work showed that glucagon‐like peptide 2 enhances lacteal contractility and chylomicron transport through VEGFR3‐ and MLC2‐dependent signaling [135, 136]. At the endothelial level, chylomicron‐derived lipids actively promote lacteal junction opening through ROCK‐dependent cytoskeletal contraction, whereas VEGFA/VEGFR2–VEGFR3–PI3K/AKT signaling restrains this response by limiting ROCK‐mediated contractility [89]. Thus, lacteal performance depends on coordinated junctional state, local contractility, and downstream drainage. This state dependence is further reinforced by regional and inflammatory remodeling, with junctional zippering reducing absorptive efficiency under pathological conditions [57].

This absorptive state also requires continuous niche support. Delta‐like ligand 4 (DLL4)‐Notch signaling is required for adult lacteal regeneration, and endothelial‐specific *Dll4* deletion causes lacteal shortening and markedly reduces postprandial plasma chylomicron levels [88]. VEGFA/VEGFR2 signaling promotes button‐to‐zipper remodeling; this restricts chylomicron uptake and protects against diet‐induced obesity, but shifts lacteals toward a less permissive absorptive state [87]. By contrast, macrophage‐derived VEGFC preserves lacteal integrity through VEGFR3, whereas antibiotic treatment or VEGFC deficiency shortens lacteals and impairs chylomicron transport [137]. Stromal fibroblast subsets also support lacteal integrity through Yes‐associated protein/transcriptional coactivator with PDZ‐binding motif‐induced VEGFC, while ROCK‐ and VEGFA/VEGFR2–VEGFR3–PI3K/AKT‐dependent signals tune lacteal permeability [57, 89, 138]. Together, these findings suggest that lacteal‐associated LEC states are continuously sustained and remodeled by microbiota‐, stromal‐, immune‐, and growth factor‐derived cues rather than governed by a single trophic pathway.

The significance of this route extends beyond intestinal fat absorption itself. Earlier studies established that intact lymphatic transport is also required for reverse cholesterol transport, such that impaired lymphatic function reduces high‐density lipoprotein (HDL)‐dependent movement of cholesterol from peripheral tissues toward the circulation [139, 140]. Lacteal or downstream drainage abnormalities may therefore affect both chylomicron uptake and systemic lipid handling. Seen in this way, lacteal‐associated LECs do not simply participate in a local absorptive process. Through region‐specific organization and state‐dependent control of junctional configuration, contractile coupling, and trophic maintenance, they shape the efficiency of intestinal lipid transport and, in turn, influence whole‐body metabolic physiology [57, 141].

### 3.3 LECs at the Immune Interface: Cell Trafficking, Antigen Handling, and Peripheral Tolerance

Beyond fluid and lipid transport, LECs form a selective immune interface along the tissue‐to‐node axis. This is particularly evident in lymph nodes, where distinct LEC subsets not only regulate the entry and positioning of antigen‐bearing DCs, but also shape how antigens are retained, redistributed, and interpreted by T cells [5, 16, 17, 142]. Immune control along the lymphatic axis is therefore best understood as a site‐ and state‐dependent process in which different LEC programs coordinate cell trafficking, antigen handling, and peripheral tolerance [5, 130, 143] (Figure 2C).

#### 3.3.1 LECs Direct Immune Cell Entry, Intranodal Routing, and Egress

The immunological influence of LECs begins before immune cells enter the lymph node parenchyma. In initial lymphatic capillaries, LECs display immobilized CCL21 together with additional adhesion‐regulating cues that support CCR7‐dependent docking and DC transmigration [144]. CD44–hyaluronan–LYVE1 interactions further stabilize leukocyte engagement with the lymphatic wall [130]. Entry is also spatially selective rather than stochastic. DCs preferentially access lymphatic capillaries through preformed portals associated with basement membrane discontinuities and multicellular endothelial junctions, where localized CCL21 exocytosis helps direct transmigration [83, 145]. Once inside the vessel, they continue to migrate along intralymphatic CCL21 gradients toward downstream collectors and, ultimately, draining lymph nodes, while chemokine scavenging and proteolytic remodeling further tune gradient structure and migratory efficiency [73, 144, 146, 147]. Afferent lymphatic transport is therefore not passive cell passage through an open conduit, but a stepwise trafficking program actively organized by LEC‐derived adhesive, chemotactic, and positional cues.

Inflammation does not simply make this route more permissive; it reconfigures it. Tumor necrosis factor α (TNF‐α) activates LECs and induces intercellular adhesion molecule 1 (ICAM1) and vascular cell adhesion molecule 1 (VCAM1), thereby enhancing DC adhesion and transendothelial migration [148]. Inflammatory remodeling also preferentially affects collectors, where VCAM1 upregulation supports rapid DC entry and delivery to draining lymph nodes [149]. Yet this shift alters more than transport speed. Direct contact with inflamed lymphatic endothelium can restrain DC maturation through ICAM1–macrophage‐1 antigen (MAC1) interactions and reduce subsequent T‐cell stimulatory capacity in the absence of pathogen‐derived cues, while transit through afferent lymphatics may itself impose a tolerogenic imprint on migrating DCs [143, 150]. Inflammatory lymphatics therefore modify both the efficiency and the immunological meaning of passage.

A similar active logic governs cell positioning after arrival in the node. Within the subcapsular sinus, ACKR4/CCRL1 scavenges CCR7 ligands and helps establish a physiological CCL21 gradient across the sinus; together with the specialized architecture of the sinus floor, this gradient directs afferent lymph‐borne CCR7^+^ DCs from the sinus into the nodal parenchyma [14, 75]. LECs thus do not merely partition nodal space structurally, but render that space functionally instructive.

This spatial control extends to immune‐cell exit as well. Lymphocyte egress depends on S1P receptor 1 (S1PR1) signaling, and LECs establish the directional basis for this process by releasing S1P into the sinus lumen and lymph through spinster homolog 2 (SPNS2). Loss of *Spns2* reduces lymph S1P, disrupts egress‐promoting gradients, and impairs T‐ and B‐cell exit, contributing to peripheral lymphopenia [151, 152, 153]. Within lymph nodes, local S1P niches also support medullary NK‐cell positioning and provide survival signals to naive T cells [154, 155]. Imaging studies suggest that egress is a multistep process involving cortical sinus probing, S1PR1‐dependent entry, and flow‐mediated progression, whereas LEC‐intrinsic programs can further tune local S1P availability and lymphocyte persistence [107, 156]. In addition, LEC‐derived programmed death‐ligand 1 (PD‐L1) can regulate the kinetics of T‐cell transendothelial migration, including that of activated regulatory T cells and CD4 effector T cells [157]. Together, these findings show that LECs coordinate immune trafficking through entry, routing, positioning, and exit rather than merely lining the route.

#### 3.3.2 LECs Shape Antigen Entry, Archiving, and Reuse Within Lymph Nodes

LECs also shape immunity by controlling not only which cells move through lymph nodes, but also how antigens enter, persist, and re‐emerge within them. Antigen entry is not homogeneous. Low‐molecular‐weight antigens can access the fibroblastic reticular cell (FRC) conduit system beneath the subcapsular sinus and are rapidly delivered to the T‐cell zone and follicles, with a fraction reaching high endothelial venule‐associated conduit and perivascular regions [158, 159, 160]. By contrast, particulate or high‐molecular‐weight antigens, as well as pathogens, tend to remain at the luminal face of the subcapsular sinus, where they are captured by CD169 macrophages at the sinus‐follicle border and relayed to follicular compartments [161, 162, 163]. Although multiple stromal and immune populations participate in this sorting process, these routes are established within an entry and retention architecture in which LECs provide an organizing interface. The lymph node is therefore not simply a site of antigen arrival, but a structured filtering system in which LECs help determine how incoming material is spatially distributed and retained.

Beyond spatial sorting, LECs can directly acquire and archive antigen. Earlier work showed that this archived material can persist for several weeks [77], whereas later quantitative and single‐cell analyses suggested that caveolae‐mediated endocytosis contributes to antigen uptake and retention [5, 81, 164]. Thus, LECs do not simply permit antigen transit through nodal space, but can act as reservoirs that extend local immune surveillance.

Archived antigen is also not biologically inert. During the refractory phase of the immune response, migratory conventional DCs can acquire antigens from LECs and cross‐present them, thereby prolonging the window of cluster of differentiation 8‐positive (CD8^+^) T‐cell stimulation; in the setting of viral antigen archiving, this process is mediated primarily by BATF3‐dependent type 1 conventional DCs (cDC1s) and is linked to LEC apoptosis [76]. Likewise, heterologous innate stimulation, including viral infection or cytosine–phosphate–guanine exposure, can mobilize archived antigens and enhance local reactivation of memory CD8^+^ T cells [164]. More recent work further indicates that antigen archiving is associated with a defined transcriptional program and preferentially localized to particular LEC subsets [5]. LECs therefore regulate not only antigen access to the node, but also its persistence, redistribution, and reuse over time, with direct implications for the duration and recall of local immune responses [5, 76, 77, 81, 164].

#### 3.3.3 LEC‐Mediated Antigen Presentation and Peripheral Tolerance

LEC‐mediated immune regulation is also evident in antigen presentation and T‐cell fate. Along the major histocompatibility complex Class I (MHC I)–CD8+ T‐cell axis, LECs constitutively express MHC I and can directly present peripheral tissue antigens to CD8^+^ T cells [17]. Under limited costimulation and high PD‐L1 expression, this interaction favors clonal deletion or functional inactivation rather than effector differentiation [16]. Programmed cell death protein 1 (PD‐1) upregulation on T cells further limits access to high‐affinity IL‐2 receptor signaling, and blockade of PD‐1/PD‐L1 or supplementation with exogenous costimulation can partially rescue these T‐cell clones [16]. Consistently, lymphatic‐specific PD‐L1 deletion expands tumor‐specific CD8^+^ T cells in tumor‐draining lymph nodes, largely by reducing apoptosis of tumor‐specific central memory T cells [98]. Tumor‐draining lymph nodes also harbor PD‐1/PD‐L1‐responsive tumor‐specific memory CD8+ T‐cell populations, and LEC PD‐L1 can regulate T‐cell behavior at the lymphatic interface [157, 165]. The MHC I–CD8 arm of LEC biology therefore supports a direct and cell‐source‐specific mechanism of peripheral restraint.

This logic is not mirrored directly along the MHC II–CD4^+^ T‐cell axis. LECs express MHC II, and inflammatory conditions can further increase this expression; lymph node stromal cells, including LECs, can acquire peptide–MHC II complexes from DCs through cross‐dressing [142, 166, 167]. In this context, stromal MHC II presentation can reduce the survival and proliferation of antigen‐specific CD4^+^ T cells and promote hyporesponsiveness [167]. Moreover, LEC MHC II can cooperate with lymphocyte activation gene 3 and the PD‐1/PD‐L1 axis to reinforce deletional tolerance in CD8^+^ T cells, although steady‐state LECs do not efficiently perform classical endogenous MHC II‐mediated presentation to CD4^+^ T cells [142]. The MHC I and MHC II arms of LEC‐mediated regulation are therefore mechanistically distinct, even if both converge on tolerance‐promoting outcomes.

LECs also indirectly shape these outcomes by altering the stromal conditions under which antigen presentation is interpreted. TNF‐α induces ICAM1 on LECs, and ICAM1–MAC1 engagement suppresses DC maturation and costimulatory molecule upregulation, thereby reducing antigen‐presenting capacity [150]. Under interferon‐γ (IFN‐γ) stimulation, human lymph node LECs can activate inhibitory pathways such as indoleamine 2,3‐dioxygenase, further restricting CD4^+^ T‐cell proliferation [168]. The tolerogenic influence of LECs thus extends beyond the peptide–MHC synapse itself into the broader stromal immune environment.

However, LEC‐mediated outcomes are not uniformly suppressive. Under steady‐state conditions, LECs can also generate a memory‐like CD8^+^ population that remains capable of reactivation, and upon antigen re‐encounter in an inflammatory setting these cells expand rapidly and acquire effector functions [169]. LEC‐mediated antigen presentation is therefore context dependent, with deletion, restraint, and recall shaped by inflammatory and stromal cues [98, 157, 169].

#### 3.3.4 LEC Heterogeneity and Site‐Specific Immune Specialization

The coexistence of trafficking control, antigen archiving, and tolerance induction reflects the compartmental organization of lymph node LECs. Single‐cell and spatial transcriptomic studies have identified multiple subsets, including cLECs in the ceiling of the subcapsular sinus, fLECs in the floor of the subcapsular sinus, and distinct cortical and medullary sinus LEC populations, each occupying a defined anatomical niche and displaying characteristic transcriptional programs [14, 15]. These subsets are not functionally interchangeable: their contributions to inflow, routing, antigen handling, and outflow are position dependent and embedded within nodal architecture.

This anatomical segregation is mirrored by molecular specialization. cLECs are enriched for programs that shape chemokine availability and intranodal routing [14, 73, 75], whereas other sinus‐associated subsets align more closely with antigen handling, cellular exit, and recirculatory control [14, 15, 170]. Thus, nodal LEC heterogeneity explains how distinct immune functions are partitioned across discrete lymphatic microdomains.

This organization also influences how the lymphatic immune interface responds to inflammatory stress. During chikungunya virus infection, viral RNA accumulates preferentially in MARCO‐associated LEC populations and is accompanied by rapid remodeling of lymph node LEC composition and function [171]. Single‐cell studies likewise indicate that inflammatory insults reshape lymph node LEC subset composition and state, as observed in settings such as skin inflammation [6]. Thus, immune‐site specialization determines not only steady‐state function, but also which LEC subsets are preferentially reprogrammed under stress.

Nor is this site specificity confined to lymph nodes. Across organs, LEC states are further shaped by local barrier exposure, drainage demand, and tissue‐specific environmental cues. The identification of meningeal lymphatics, in particular, clarified an important anatomical and functional link between central and peripheral immunity [96]. Taken together, these observations indicate that the lymphatic immune interface is built from localized LEC programs rather than from a uniform endothelial lining.

## 4 Lymphatic Dysfunction in Disease

LEC dysfunction in disease is best understood as context‐dependent failure of lymphatic functional modules, rather than as a uniform change in vessel abundance. Core programs controlling network assembly and repair, fluid uptake and transport, immune coordination, barrier regulation, lipid handling, and clearance–immune coupling may destabilize in different combinations according to developmental stage, tissue context, and disease pressure [96, 172, 173, 174]. Thus, similar lymphatic changes, including expansion, dilation, or regression, may produce divergent biological outcomes across diseases. Accordingly, this section is organized by dominant pathological modes: failed lymphatic network formation or repair [63, 106, 175, 176], maladaptive inflammatory and immune‐regulatory remodeling [172, 177, 178], tumor‐associated route expansion and immune suppression [98, 179], and coupled drainage–lipid–immune dysfunction in metabolic or cardiovascular disease [173, 174, 180, 181]. These disease settings are discussed as representative contexts in which specific LEC functions are disrupted and contribute to tissue pathology, rather than as isolated organ‐specific categories (Table 2).

**TABLE 2:** Disease‐context‐dependent modes of LEC dysfunction and dominant consequences.

Disease Setting	Dominant defect	Affected LEC function	Consequences	Therapeutic implication	References
Primary lymphatic disease	Developmental failure	Network assembly; valves; one‐way transport	Early drainage failure; unstable transport architecture	Restore assembly and transport competence	[46, 182, 183, 184, 185]
Secondary lymphedema	Failed repair and progressive decompensation	Fluid uptake; collectors; valves; regeneration	Persistent edema; fibrosis; fat deposition	Promote repair and niche reconstruction	[186, 187, 188, 189, 190, 191]
Chronic inflammation and autoimmunity	Maladaptive immune remodeling	Cell trafficking; chemokine shaping; antigen handling	Inflammatory retention; unresolved immune activation	Reprogram immune‐interface functions	[6, 16, 192, 193]
Tumor progression and lymphatic metastasis	Metastasis‐supporting and immunosuppressive reprogramming	Lymphangiogenic expansion; barrier regulation; immune suppression; tumor–LEC crosstalk	Nodal metastasis; local immune suppression	Restrain remodeling and reverse immune suppression	[98, 194, 195, 196]
Metabolic and cardiovascular disease	Coupled failure of drainage, lipid handling, and immune homeostasis	Clearance; lipid transport; immune egress; repair	Impaired RCT; persistent inflammation; adverse remodeling	Restore drainage–metabolism–immune coupling	[91, 139, 140, 174, 180, 197, 198, 199, 200]
Meningeal lymphatic dysfunction in CNS disease	Clearance–immune coupling failure	CNS efflux; immune surveillance; neuroimmune communication	Impaired clearance; altered neuroinflammation	Define whether drainage should be restored or restrained	[93, 94, 95, 96]

*Abbreviations*: CNS, central nervous system; LEC, lymphatic endothelial cell; RCT, reverse cholesterol transport.

### 4.1 Lymphatic Disease as Developmental Disruption and Failed Repair

Hereditary and acquired lymphatic disorders illustrate two major routes to lymphatic failure: developmental disruption and failed repair [46, 176, 201, 202]. In primary disease, these abnormalities are usually embedded during development and determine whether a functional lymphatic network can be established, whether collecting vessels can mature appropriately, and whether valves can support stable transport [46, 203, 204]. In secondary disease, by contrast, the central problem arises after injury, when a lymphatic system that initially retains partial compensatory capacity fails to repair effectively and enters chronic structural decompensation driven by persistent stasis, inflammation, and fibrosis [176, 204]. Thus, although both settings may converge clinically as inadequate drainage, they differ in when and how LEC functions become destabilized.

#### 4.1.1 Primary Lymphedema: Developmental Failure of the Lymphatic System

Primary lymphedema is best understood not simply as early‐onset swelling or as a uniform deficit in lymphatic vessel number, but as a heterogeneous developmental disorder in which distinct LEC programs fail at different levels of transport‐system formation [44, 46, 203, 205]. These defects can affect lymphatic fate specification and network assembly, collector‐vessel and valve maturation, or the flow‐dependent mechanisms that stabilize unidirectional transport. Disorders that converge clinically as impaired drainage may therefore differ substantially in both molecular origin and structural basis [182, 206]. Milroy disease remains the clearest example of failure at the level of network establishment: *FLT4* mutations impair VEGFR3 kinase activity and directly link disruption of the VEGFC–VEGFR3 axis to defective lymphatic network formation and maintenance [185, 206]. Yet this pathway is not functionally isolated. VEGFR3 signaling is embedded within broader developmental circuitry, including Ang2/Tie/PI3K‐dependent maintenance of cell‐surface VEGFR3 and TIE1‐dependent lymphatic remodeling [46, 49, 182]. These studies also suggest that some underdeveloped networks retain limited reconstructive potential, although rescue through VEGFC remains strongly dose‐ and context‐dependent because excessive signaling can induce vascular dilation and altered permeability [175, 207, 208, 209]. Primary lymphedema should therefore not be framed simply as a disease of insufficient lymphangiogenesis.

A second developmental layer becomes evident in *FOXC2*‐ and *GATA2*‐associated disease, which highlights failure not of initial network establishment, but of collector‐valve maturation and stable one‐way transport [44, 182]. FOXC2 is strongly linked to lymphedema‐distichiasis syndrome [210, 211], and mechanistic studies show that FOXC2 cooperates with nuclear factor of activated T cells 1 (NFATC1) to promote collecting‐vessel and valve maturation, while PROX1/FOXC2‐dependent fluid‐shear signaling contributes to valve precursor formation [44, 47, 212]. *GATA2*‐associated Emberger syndrome extends this logic by showing that GATA2 is required for lymphatic valve formation and maintenance [48, 213]. In these disorders, the dominant defect lies less in absence of capillary lymphatics than in failure of the collector‐valve unit to achieve coordinated structural and functional maturity. Clinically, this distinction matters because progressive worsening reflects not only early edema, but also the cumulative effects of valvular reflux, recurrent cellulitis, and tissue fibrosis [214].

Primary lymphedema also extends beyond lymphatic hypoplasia and valve immaturity into defects in intercellular communication, mechanosensation, ligand maturation, morphogenesis, and proliferative control [44, 215, 216]. *GJC2* mutations suggest that disturbed gap‐junction communication may impair transmission of flow‐related signals [217, 218], whereas *PIEZO1* mutations place mechanosensitive ion channels within the pathogenic spectrum of lymphatic dysplasia [44, 205, 219]. Mutations in *CCBE1* and *FAT4*, as seen in Hennekam syndrome, further show how defects in ligand maturation and morphogenesis can produce widespread lymphatic hypoplasia [220, 221], while *SOX18* and *KIF11*‐related syndromes underscore the contribution of fate specification and proliferative control to network formation [215, 222, 223]. Together, these disorders show that primary lymphedema is not a single developmental entity, but a mechanistically heterogeneous group in which LEC programs fail at different anatomical and functional levels of lymphatic construction and maturation [216, 224, 225, 226].

#### 4.1.2 Secondary Lymphedema: Chronic Remodeling After Lymphatic Injury

Compared with primary lymphedema, secondary lymphedema is better understood not simply as a consequence of vessel transection or outflow obstruction, but as failed repair that progresses into chronic tissue remodeling after lymphatic injury. Here, the central problem is not injury alone, but failure of the lymphatic system and surrounding tissue niche to regain functional recovery. Injured tissues instead enter a self‐reinforcing cycle in which stasis, inflammation, fibrosis, and fat deposition amplify one another, converting an initially reversible transport defect into structural decompensation [204, 227, 228]. Breast cancer‐related lymphedema is among the most common treatment‐associated examples, with incidence estimates of 16.6–21.9% and risk influenced by higher body mass index, axillary lymph node dissection, and radiotherapy [229, 230]. Yet these epidemiological factors define susceptibility more readily than mechanism and do not explain why only a subset of patients progress from injury to persistent inflammation, functional decline, and chronic tissue remodeling [190, 204, 231]. Secondary lymphedema is therefore more appropriately framed as a chronic inflammatory and remodeling disorder than as a purely mechanical consequence of lymphatic stasis [232, 233].

Experimental studies support this view by showing that, once established, secondary lymphedema behaves as a self‐sustaining pathological state rather than a transient transport defect. In mouse models, CD4^+^ T cells are required for disease development; activated in regional draining lymph nodes, they migrate to injured skin and are associated with increased fibrosis, suppressed lymphangiogenesis, and impaired lymphatic function [189]. More recent work further suggests that this CD4^+^ T‐cell response may be antigen driven, with oligoclonal expansion detected in both human lymphedema tissue and mouse disease models [190]. TGF‐β1 acts as a key profibrotic driver by promoting extracellular matrix deposition, amplifying inflammation, and increasing tissue stiffness [191]. These findings indicate that failed immune resolution and progressive matrix remodeling are not secondary by‐products of edema, but central components of the chronic disease program.

This failed‐repair state also extends into LEC‐intrinsic reprogramming. In breast cancer‐associated secondary lymphedema, the stress‐induced lncRNA LINC–PINT is highly expressed in LECs and reshapes chromatin accessibility near genes involved in lymphangiogenesis and immune‐cell adhesion; conditional loss of *Lnc–Pint* in lymphatic endothelium reduces dermal backflow, fibrosis, and inflammation [106]. This finding suggests that injured LECs can enter maladaptive state transitions that help stabilize chronic remodeling, extending the mechanism beyond extrinsic stasis and immune activation alone. As disease progresses, fat accumulation and fibrosis become persistent structural burdens, so that the dominant problem gradually shifts from impaired drainage to chronic tissue remodeling [228, 234]. At that stage, restoration of lymphatic outflow often yields only limited benefit, because dysfunction is no longer confined to fluid conduits but is embedded in the altered tissue niche and in the reduced responsiveness of the residual lymphatic network [234, 235].

Clinical intervention studies provide partial support for this stage‐dependent model, but also highlight its therapeutic limits. Ketoprofen reduced skin thickness and improved histological parameters in an exploratory lymphedema study, whereas Lymfactin combined with vascularized lymph node transplantation showed feasibility and signals of improvement in Phase I follow‐up but no significant advantage over placebo on the 12‐month primary endpoint in a subsequent Phase II trial [236, 237, 238, 239, 240]. These findings suggest that lymphangiogenesis‐promoting strategies are likely to be strongly stage dependent and that current outcome measures may be insufficiently sensitive to detect early functional recovery [241, 242]. Future work will therefore need tighter alignment among intervention type, disease stage, patient stratification, and endpoint selection.

Infectious secondary lymphedema provides a further reminder that similar edema phenotypes can arise from distinct pathogenic routes. Filarial lymphedema has traditionally been approached through parasite burden, host immune responses, and basic care, whereas direct LEC‐centered mechanistic evidence remains comparatively limited [243]. Randomized trials suggest that doxycycline can improve mild‐to‐moderate filarial lymphedema, potentially independently of current infection status [244]. However, a double‐blind randomized trial in India found no clear additional benefit of doxycycline when a standardized essential package of care was fully implemented [245]. These findings suggest that basic care itself confers substantial benefit, whereas any incremental effect of doxycycline is likely conditioned by disease stage, pathogen background, and adherence to care [246]. Secondary lymphedema should therefore not be treated as a single biological mechanism with uniform therapeutic logic, but as a shared clinical endpoint reached through context‐dependent pathways of failed repair.

### 4.2 Lymphatic Remodeling in Inflammation and Autoimmunity

In inflammatory and autoimmune diseases, lymphatic remodeling is not adequately explained as passive vessel expansion secondary to established inflammation. This view captures part of the phenotype, but it cannot explain why similar structural changes have divergent functional consequences across tissues and disease stages. The key issue is which LEC populations are remodeled, how altered states affect drainage, immune‐cell trafficking, antigen transport, and local tissue homeostasis, and when these shifts remain compensatory or become maladaptive [177, 247, 248]. This perspective is increasingly supported by subset‐specific responses in peripheral tissue inflammation and rapid changes in lymph node LEC composition during chikungunya virus infection [6, 171]. Inflammatory lymphatic remodeling should therefore be viewed as context‐dependent LEC reprogramming that can support resolution, adaptation, or disease persistence.

#### 4.2.1 Inflammatory Bowel Disease: Early Lymphatic Alteration and Remodeling

In inflammatory bowel disease (IBD), lymphatic abnormalities can emerge early rather than only after chronic inflammation is established [249]. Intraoperative observations by Heatley et al. revealed abnormal mesenteric lymphatic drainage even in intestinal segments without grossly visible lesions in Crohn's disease, suggesting that lymphatic dysfunction is not simply a late consequence of overt mucosal injury [250]. Subsequent human histological studies described lymphocytic perilymphangitis, lymphocytic or granulomatous lymphatic obstruction, and increased lymphatic vessel density or dilation in IBD tissue [251, 252, 253, 254]. However, structural changes alone do not establish whether remodeling is adaptive, dysfunctional, or stage dependent.

Functional studies make clear that lymphatic alterations in IBD must be interpreted by disease stage and inflammatory context [248]. Rahier et al. found that lower lymphatic vessel density in the mucosa and submucosa of the proximal ileal resection margin in Crohn's disease was associated with endoscopic recurrence within 1 year after surgery, suggesting that preservation of local lymphatic capacity may help sustain remission [255]. Experimental studies further show that the consequences of augmenting lymphangiogenic signaling are not uniform. D'Alessio et al. showed that VEGFC‐mediated enhancement of lymphatic function in acute and chronic colitis models reduced inflammation and tissue damage, improved trafficking of inflammatory cells to draining lymph nodes, and promoted clearance of bacterial antigens [256]. By contrast, Wang et al. reported that VEGFC overexpression aggravated epithelial injury, edema, and neutrophil infiltration in an acute dextran sulfate sodium (DSS) colitis model [257]. Consistent with a protective role for baseline lymphatic activity, Jurisic et al. found in *IL10*‐deficient mice that VEGFR3 blockade worsened colitis and was accompanied by more severe lymphangiectasia, vessel twisting, and submucosal edema, further underscoring the importance of VEGFR3‐dependent lymphatic function in limiting inflammation [258].

Overall, intestinal lymphatic remodeling in IBD is stage and state dependent: it may support inflammatory clearance when functional drainage is preserved, but contribute to edema, barrier disruption, and chronic inflammation when remodeling becomes maladaptive.

#### 4.2.2 Skin and Joint Inflammation: Functional Remodeling of the Lymphatic Response

In chronic inflammatory skin disease, lymphatic remodeling is best viewed as an active but functionally variable response to inflammation. Henno et al. found that vascular dilation is already prominent in early pinhead lesions, whereas lymphatic dilation and proliferation become more evident at the mature plaque stage, linking lymphatic remodeling more closely to persistent inflammation and cumulative tissue burden than to early disease initiation [259]. Moustou et al. further showed that expression of D2‐40, VEGFC, and vascular endothelial growth factor D (VEGFD) is increased in psoriatic lesions, and that VEGFC expression on lymphatic vessels decreases after etanercept treatment, suggesting that at least part of this remodeling program remains reversible with systemic anti‐inflammatory therapy [260]. Functional studies reinforce this view: impaired lymphatic transport aggravated psoriasis‐like skin inflammation in mice through accumulation of inflammatory cytokines [261], whereas direct evidence for dynamic lymphatic function in human psoriasis remains limited, despite measurable microlymphatic abnormalities in plaque and perilesional skin [262].

The biological consequence of this remodeling depends less on vessel expansion than on restoration of effective drainage. Huggenberger et al. showed in a K14–VEGFA chronic dermatitis model that sustained VEGFR3 activation enhances lymphatic drainage and alleviates inflammation, indicating that recovery of functional outflow, rather than expansion of lymphatic area per se, may be the decisive variable in this setting [263]. In a K14–IL4 atopic dermatitis model, Shi et al. observed enhanced dermal lymphangiogenesis in the context of Th2 inflammation, accompanied by macrophage recruitment and upregulation of the VEGFC–VEGFR3 axis; however, these changes paralleled disease severity and did not by themselves establish that lymphatic remodeling was sufficient to confer anti‐inflammatory benefit [264]. Lymphatic alterations in psoriasis and atopic dermatitis are therefore best understood as adaptive but not uniformly effective remodeling responses, whose consequences depend on whether inflammatory remodeling restores functional drainage [261].

Evidence in joint disease remains much weaker. In psoriatic arthritis, direct studies focused on LECs remain limited, and most available evidence comes from case reports or small series suggesting that a subset of patients may exhibit lymphedema or abnormal lymphatic drainage, with lymphoscintigraphy revealing absent or impaired transport in some cases [265]. These observations indicate that the lymphatic system is not irrelevant to PsA, but they remain insufficient to define whether joint lymphatic remodeling is compensatory, maladaptive, or mechanistically linked to disease persistence. Thus, functional models derived from psoriatic skin should not be directly extrapolated to inflamed joints.

#### 4.2.3 Rheumatoid Arthritis and Connective Tissue Disease: From Compensation to Failure

Rheumatoid arthritis (RA) provides one of the clearest examples of how inflammatory lymphatic remodeling shifts from early compensation to later functional failure. In TNF‐transgenic and serum‐induced arthritis models, TNF‐driven VEGFC expression by osteoclast precursors increased intra‐articular lymphangiogenesis, whereas VEGFR3 blockade reduced joint and nodal lymphangiogenesis, decreased drainage, and worsened synovial inflammation [266, 267]. These findings indicate that the VEGFC–VEGFR3 axis supports a compensatory lymphatic response in chronic arthritis. However, this compensatory phase is not sustained. Liang et al. further showed that, in TNF‐Tg inflammatory arthritis, LECs express high levels of inducible nitric oxide synthase (iNOS), which suppresses lymphatic smooth muscle contraction, impairs collecting‐vessel pump function, and reduces drainage efficiency [247, 268]. Thus, RA lymphatic remodeling shifts from early expansion to transport failure when inflammation disables the contractile transport apparatus [247].

This model shifts attention from lymphatic expansion alone to restoration of contraction and drainage [269, 270, 271]. Bouta et al. found that anti‐TNF treatment improves lymphatic contractility and drainage in TNF‐driven arthritis, suggesting that suppression of inflammation can partially restore lymphatic function [272]. Manzo et al. further reported that axillary draining lymph node ultrasound changes in RA correlate with peripheral joint activity and response to anti‐TNF therapy, raising the possibility of a noninvasive window into lymphatic‐related function [273, 274]. Even so, these measurements cannot yet be regarded as direct readouts of LEC function, and their clinical sensitivity and specificity remain to be established in larger cohorts.

Among connective tissue diseases, systemic sclerosis shows a different pattern, in which lymphatic depletion appears more prominent than compensation. Human studies showed that dermal lymphatic vessels are reduced in SSc skin, with progressive loss in advanced disease, suggesting that the transition from early edema to late fibrosis is accompanied by continuing attrition of the lymphatic network [275, 276]. Other studies further link lymphatic scarcity to increased risk of digital ulcers, implying that loss of LEC‐supported drainage may aggravate peripheral circulatory compromise [277]. A 2024 study implicating ERG/FLI1‐related transcriptional regulation provides additional molecular support for lymphatic depletion in SSc [177]. However, available data do not yet resolve whether fibrosis drives lymphatic collapse or whether early lymphatic dysfunction contributes to fibrotic progression [278].

Evidence in systemic lupus erythematosus points to a third pattern, in which the significance of lymphatic remodeling diverges across organs. Howlader et al. found that impaired lymphatic flow from skin to draining lymph nodes is associated with photosensitive dermatitis and enhanced B‐cell responses in the draining node; in mouse models, improving lymphatic flow attenuated photosensitivity, suppressed lymph node immune activation, and influenced the FRC/monocyte regulatory axis [279]. By contrast, Wang et al. reported in a lupus nephritis model that VEGFR3 inhibition reduces renal lymphangiogenesis and alleviates renal inflammation, suggesting that lymphatic remodeling does not carry the same biological meaning in skin and kidney even within the same systemic disease [280]. Overall, connective tissue diseases show divergent lymphatic phenotypes rather than a single protective or pathogenic pattern: in RA, compensatory expansion can progress to transport failure; in SSc, depletion predominates; and in SLE, effects diverge by organ context.

### 4.3 LEC Reprogramming in Tumor Lymphatic Metastasis

**FIGURE 3:**
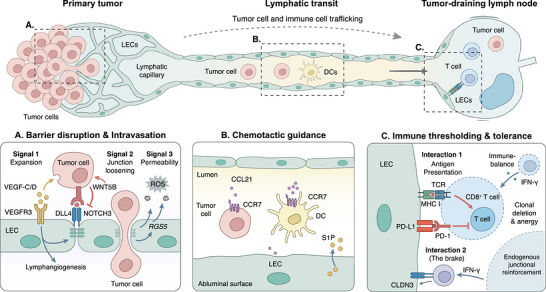
Tumor‐associated reprogramming of lymphatic endothelial cells. (A) In primary tumors, VEGFC/VEGFD–VEGFR3 signaling promotes lymphatic expansion, whereas DLL4–NOTCH3, WNT5B, ROS, and RGS5‐associated junctional remodeling increase lymphatic barrier permissiveness and support tumor‐cell intravasation. (B) During lymphatic transit, LEC‐associated CCL21 cues engage CCR7 on tumor cells and dendritic cells, whereas S1P‐related signals may provide additional directional support along lymphatic routes. (C) In tumor‐draining lymph nodes, LECs impose immune thresholding and tolerance through antigen presentation and PD‐L1–PD‐1 signaling, while IFN‐γ‐associated feedback and CLDN3‐related junctional responses reflect an attempt to preserve barrier integrity. CCL21, C–C motif chemokine ligand 21; CCR7, C–C motif chemokine receptor 7; CLDN3, claudin 3; DC, dendritic cell; DLL4, delta‐like ligand 4; IFN‐γ, interferon‐γ; LEC, lymphatic endothelial cell; MHC I, major histocompatibility complex Class I; NOTCH3, Notch receptor 3; PD‐1, programmed cell death protein 1; PD‐L1, programmed death‐ligand 1; RGS5, regulator of G protein signaling 5; ROS, reactive oxygen species; S1P, sphingosine‐1‐phosphate; TCR, T‐cell receptor; VEGFC/VEGFD, vascular endothelial growth factor C/D; VEGFR3, vascular endothelial growth factor receptor 3; WNT5B, Wnt family member 5B.

In tumors, LECs are not simply anatomical conduits for lymphatic spread. Rather, tumor progression is accompanied by reprogramming of LEC states that reshape lymphatic dissemination, barrier function, and immune control [97, 281, 282, 283]. Clinical and pathological studies have long shown that increased intratumoral and peritumoral lymphatic vessel density is frequently associated with greater sentinel and regional lymph node metastasis, as well as poorer prognosis [284, 285, 286, 287, 288, 289]. Yet these correlations cannot be explained solely by greater vessel availability. Tumors reconfigure not only lymphatic abundance, but also lymphatic function (Figure 3).

Classically, this reprogramming includes lymphangiogenic expansion through the VEGFC/VEGFD–VEGFR3 axis and, in some contexts, VEGFR2‐related signaling [290, 291, 292, 293, 294, 295, 296] (Figure 3A). However, tumor‐associated lymphatic remodeling is not simply a quantitative increase in vessel number. More fundamentally, it shifts lymphatic function away from tissue drainage and immune surveillance and toward dissemination and immune evasion. Tumor lymphatics are therefore better understood as a reprogrammed vascular compartment that favors tumor‐cell entry, transit, and metastatic seeding rather than homeostatic clearance [97, 282, 283].

Immune reprogramming is another major component of this state shift. Tumoral LECs can acquire MHC Class II‐restricted antigen‐presenting capacity and act locally within the tumor microenvironment to reinforce regulatory T‐cell suppressive function and dampen effector T‐cell immunity [97, 194]. In parallel, tumors and LECs cooperate to generate directional cues, including the CCL21–CCR7 axis and S1P, that guide both tumor cells and immune cells along lymphatic routes [146, 297, 298, 299, 300, 301] (Figure 3B). In tumor‐draining lymph nodes, LEC autophagy further regulates intranodal lymphocyte positioning, T‐cell egress, recruitment of tumor‐infiltrating T and NK cells, and responsiveness to immune checkpoint blockade by modulating S1P availability [107, 302]. Lymph node LECs also express high levels of PD‐L1 and support antigen uptake, MHC I presentation, and cross‐presentation. In tumor settings, these programs can induce deletion or functional inactivation of CD8^+^ T‐cell clones [16, 98], support prolonged antigen archiving [77], and, in melanoma, enhance LEC‐mediated cross‐presentation that promotes elimination of tumor‐specific CD8^+^ T cells [303]. Thus, the contribution of LECs to tumor lymphatic metastasis extends beyond tumor‐cell entry to tumor‐conditioned antigen handling and local immune restraint [97, 98, 107, 303] (Figure 3C).

The form of this reprogramming varies across solid tumors. In skin and mucosal tumors, reciprocal tumor–LEC signaling is prominent. DLL4 expressed by LECs activates NOTCH3 in tumor cells and upregulates WNT5B; tumor‐derived WNT5B then feeds back to weaken LEC tight junctions, increase endothelial permeability, and cooperate with MMP14 and β1‐integrin to promote transendothelial migration. This DLL4–NOTCH3–WNT5B axis is closely associated with NOTCH3/WNT5B coexpression, increased lymphatic density, and poor survival [304]. In head and neck squamous cell carcinoma, tumor‐derived CXCL5 enhances LEC activation, lumen formation, and barrier disruption through CXCR2 signaling in LECs, thereby driving lymphatic invasion; in xenograft models, CXCR2 inhibition significantly reduces lymph node tumor burden [195]. These findings show that LECs participate in reciprocal prometastatic circuits rather than serving as passive stromal elements [281, 282, 305].

A second mode is more evident in breast, gynecological, and gastrointestinal malignancies, where LEC reprogramming is characterized more strongly by barrier destabilization, metabolic rewiring, and sustained growth‐associated signaling. In breast cancer, an oxidative stress‐responsive RGS5^+^ LEC population has been identified, and tumor‐derived reactive oxygen species can induce LEC–RGS5 expression, alter LEC metabolism, and facilitate tumor–endothelial adhesion and transendothelial migration; these changes are also linked to acquired drug resistance, whereas ROS scavenging or *Rgs5* knockdown attenuates both prometastatic and drug‐resistant phenotypes [179]. Additional tumor‐specific pathways converge on similar LEC outputs: VCAM1 upregulation in breast cancer‐associated lymphatics loosens endothelial junctions and promotes lymphatic invasion [306]; the CREB5–APLN–APLNR cascade in cervical cancer enhances LEC migration, tubule formation, and lymphangiogenesis, with APLNR inhibition reducing dissemination [307]; and IL8/CXCL1–CXCR2 signaling in gastric cancer, CXCR2–CXCL5 signaling in cholangiocarcinoma, and PROK1 in colorectal cancer support LEC growth, migration, metabolic activation, or lymphangiogenic activity linked to nodal progression [305, 308, 309, 310]. Although the initiating signals differ, these pathways converge on increased permeability, endothelial activation, metabolic rewiring, and a lymphatic state permissive for tumor transendothelial migration [311].

Prostate cancer further shows that tumor‐associated LEC reprogramming cannot be explained by the VEGFC–VEGFR3 axis alone. In addition to noncanonical activation of LEC VEGFR2 by VEGFA and VEGFC, extracellular vesicles carrying hnRNPA2B1 can drive a circPDLIM5–YY1–PROX1 signaling axis that reprograms LECs and promotes pelvic lymph node metastasis [312, 313]. Thus, tumor cells reshape LEC phenotype not only through soluble lymphangiogenic factors, but also through vesicle‐mediated intercellular signaling [313, 314].

Importantly, tumor‐associated LEC states are not irreversibly fixed in a prometastatic direction. Immune cell‐derived IFN‐γ can upregulate tight‐junction molecules such as CLDN3 in LECs while reshaping their metabolic state, thereby tightening endothelial gaps and reducing tumor penetration. In melanoma models, LEC‐specific deletion of the IFN‐γ receptor markedly increases lymphatic dissemination and lymph node metastasis, indicating that barrier normalization can function as an endogenous antimetastatic mechanism [315, 316]. These findings indicate that tumor‐associated LEC states are shaped by the balance among tumor‐derived signals, local inflammation, and immune pressure, rather than being uniformly locked into a prometastatic program [315, 316, 317].

Translationally, the key advance is the recognition that tumor‐associated LEC programs are mechanistically actionable. Existing causal evidence shows that VEGFC or VEGFD overexpression increases lymphatic vessel density and lymph node metastasis, whereas VEGFR3‐Ig, soluble VEGFR3, or blocking antibodies reduce metastatic burden by interrupting this pathway [290, 292, 293, 295, 318, 319]. Other intervention points include CXCR2 antagonism in head and neck squamous cell carcinoma, APJ inhibition in cervical cancer, and targeting of DLL4–NOTCH3–WNT5B, RGS5‐related, or EV‐circPDLIM5‐mediated LEC reprogramming [179, 195, 304, 307, 313]. Clinical and pathological studies further indicate that high lymphatic density, strong tumor expression of VEGFC and VEGFR3, and occult lymph node metastasis are associated with metastatic progression or poorer outcome, although the strength and exact nature of these associations vary across tumor types [284, 320, 321, 322, 323, 324]. Notch3/WNT5B signaling, for example, represents a more specific prometastatic LEC program rather than a general survival marker [281, 304, 325]. The next step is therefore not to suppress lymphatic vessels globally, but to distinguish tumor‐specific LEC programs that drive route expansion, immune suppression, or barrier destabilization from those that preserve lymphatic integrity and restrain dissemination.

### 4.4 Lymphatic Dysfunction Across Metabolic and Cardiovascular Diseases

In metabolic and cardiovascular disease, lymphatic dysfunction is not adequately explained as failure of a single lymphatic task. Rather, tissue‐specific LEC states that normally coordinate drainage, lipid transport, and immune homeostasis become uncoupled and maladaptively reprogrammed within diseased microenvironments. In this setting, the key issue is not simply that drainage declines, but which LEC programs are disrupted first, how this disturbs the balance among edema resolution, lipid efflux, and immune‐cell trafficking, and when these changes shift from compensation to disease‐promoting dysfunction. Reduced removal of edema fluid and inflammatory mediators, impaired lipid handling, and prolonged retention of immune cells can then reinforce one another and drive chronic tissue remodeling. Thus, metabolic and cardiovascular disease should be viewed as settings in which altered LEC states connect drainage failure with metabolic imbalance and persistent inflammation [181, 326, 327]. Recent reviews of cardiac lymphatics are consistent with this view, emphasizing that lymphatic dysfunction influences myocardial fluid balance, immune composition, collagen turnover, and lipid handling rather than merely reflecting downstream cardiovascular injury [328].

Diabetic chronic wounds provide one of the clearest examples of this integrated failure. Here, the problem is not limited to impaired perfusion or defective angiogenesis. Loss of lymphatic vessels, together with a blunted VEGFC–VEGFR3 response, restricts clearance of necrotic debris and inflammatory burden, thereby trapping wounds in a state of persistent inflammation and delayed repair [329, 330]. Brunner et al. identified reduced lymphatic vessels in chronic human ulcers, whereas targeted VEGFC delivery in db/db mice promoted lymphangiogenesis, improved wound healing, and reduced local inflammation and abnormal matrix remodeling [330]. These findings indicate that restoration of lymphatic function is itself a determinant of tissue repair rather than a secondary correlate of vascular growth [331]. What fails in diabetic chronic wounds, therefore, is not simply perfusion, but the reparative sequence through which debris is cleared, inflammation resolves, and regeneration proceeds.

Atherosclerosis places the lymphatic contribution to disease in a different mechanistic frame, because the most direct entry point is cholesterol transport. Even here, however, the lymphatic role cannot be reduced to passive drainage. Martel et al. showed that lymphatic pathways are required for macrophage reverse cholesterol transport from the arterial wall to plasma and liver and, using an aortic transplantation model, further demonstrated that impaired posttransplant lymphatic regrowth increases cholesterol retention within transplanted atherosclerotic aortic segments, thereby linking lymphatic reconnection to arterial‐wall cholesterol clearance [140]. However, this model has interpretive constraints: transplantation disrupts native lymphatic continuity and introduces surgery‐associated inflammation, immune perturbation, ischemia–reperfusion injury, and reparative lymphatic regrowth, all of which may influence graft remodeling independently of native aortic lymphatic biology [140, 332, 333, 334, 335, 336]. Mechanistic studies further demonstrated that LECs express SR‐B1 and directly support HDL transport through receptor‐dependent uptake and transcytosis, whereas interference with SR‐B1 signaling diminishes movement of labeled cholesterol through lymphatic pathways [139]. Thus, LECs contribute directly to macrophage‐derived cholesterol clearance rather than merely reflecting passive solute movement. Yet this transport‐centered view remains incomplete. Structural lymphatic expansion or dilation is not uniformly protective, because enlargement of the network may coexist with impaired drainage and persistent inflammatory‐cell retention once remodeling is no longer functionally coupled to cholesterol transport and leukocyte egress [337, 338]. More recent single‐cell and mechanistic studies further suggest that LECs themselves undergo acquired transcriptional and functional remodeling during disease progression, including altered immunoregulatory features, disruption of lipid‐handling programs such as Ldlr expression, and broader abnormalities in lipid‐transport‐associated pathways [180, 200]. Additional work likewise suggests that loss of protective pathways preserving LEC integrity and function contributes to lesion progression [339, 340].

Accordingly, lymphatic dysfunction in atherosclerosis is better understood as a coupled failure of transport competence and state regulation, through which cholesterol‐transport insufficiency and programmatic dysregulation jointly promote disease progression [180, 199, 200]. This failure is functionally relevant to plaque progression and stability, because impaired lymphatic transport and persistent adventitial inflammation are associated with lesion progression, whereas arterial VEGFC delivery improves plaque‐stabilizing features, including reduced necrotic core expansion and better preservation of the fibrous cap [199, 338]. Impaired lymphatic drainage may also sustain inflammatory‐cell retention and inflammatory‐mediator accumulation in the adventitial‐plaque microenvironment, thereby favoring protease‐associated extracellular‐matrix remodeling that weakens fibrous‐cap support and increases plaque vulnerability [341]. Beyond these classical indices of stability, unresolved inflammatory retention may also sustain a proangiogenic plaque microenvironment that favors pathological neovascularization [342, 343]; once established, these immature intraplaque microvessels are closely linked to intraplaque hemorrhage and lesion destabilization [344]. Thus, plaque progression should be interpreted by whether lymphatic remodeling remains functionally coupled to cholesterol egress, inflammatory resolution, and plaque stability. Atherosclerotic lymphatic dysfunction therefore reflects coupled failure of cholesterol efflux, inflammatory resolution, and LEC integrity rather than vessel remodeling alone.

The heart makes this drainage–inflammation–remodeling axis especially visible, because lymphatic function is directly tied to tissue edema, immune‐cell clearance, and the quality of myocardial repair. Cardiac lymphatic dysfunction alone is sufficient to induce myocardial edema, inflammation, fibrosis, and hypertrophy, ultimately leading to diastolic dysfunction [326]. Cardiac lymphatics therefore need to be regarded as an essential component of myocardial homeostasis rather than as an accessory drainage route subordinate to the blood vasculature [326]. In the aging heart, this dysfunction is marked by reduced lymphatic vessel density, vessel dilation, impaired lymphatic flow, and increased perilymphatic inflammation and fibrosis, whereas aerobic exercise partially reverses these abnormalities and improves lymphatic structure and drainage [345]. In pressure‐overload heart failure, endogenous cardiac lymphangiogenesis limits inflammatory‐cell accumulation and perivascular fibrosis while delaying left ventricular dilation and decompensation; conversely, inhibition of this response accelerates disease progression, indicating that lymphatic remodeling can remain protective in a nonischemic setting [181, 346, 347, 348]. After ischemic injury, however, the relationship between lymphangiogenesis and functional recovery is not linear. Genetic blockade of lymphatic expansion after myocardial infarction did not further reduce ejection fraction within 14 days, although it tended to increase fluid retention in the infarct region, suggesting that the benefit of postinfarction lymphangiogenesis depends on intervention strategy and observation window [349]. Even so, therapeutic studies support the broader reparative potential of this system. Preservation of LEC mitochondrial homeostasis attenuates reperfusion injury, whereas VEGFC mRNA delivery via lipid nanoparticles (LNPs) promotes lymphangiogenesis, reduces inflammatory infiltration and fibrosis, and improves postinfarction cardiac function [350, 351]. Recent single‐cell and spatiotemporal transcriptomic analyses further show that postinfarction cardiac LECs are not homogeneous, but instead segregate into subpopulations linked to metabolic stress in the infarct zone, inflammatory responses in the border zone, and edema clearance during later stages of repair [92, 197, 352]. Thus, cardiac lymphatic dysfunction after injury reflects spatially and temporally organized LEC state changes rather than drainage insufficiency alone.

A related principle is evident in heart transplantation, where restoration of lymphatic balance depends not on preservation of native vessels, but on posttransplant regeneration. Because donor lymphatic vessels are severed during transplantation and cannot be surgically reconnected, recovery of cardiac lymphatic drainage relies largely on neolymphatic reconstruction. Experimental studies suggest that these neolymphatic vessels arise mainly from recipient LYVE1^+^ cells and that activated fibroblasts promote this process through pathways including VEGFD/VEGFR3, MDK/NCL, and SEMA3C/NRP2. Inhibition of this regenerative response shortens graft survival, implying that early lymphatic reconstruction is more likely to be protective than merely proinflammatory [353]. Interpretation nevertheless requires caution, because surgical interruption of native lymphatic connections, ischemia–reperfusion injury, and alloimmune activation can each reshape lymphatic phenotype and flow, such that posttransplant remodeling cannot be directly equated with physiological lymphatic organization in the native heart [354, 355, 356]. Recent human data likewise suggest that reduced cardiac lymphatics after transplantation are associated with more severe cardiac allograft vasculopathy and poorer survival [357]. Together, these findings indicate that posttransplant cardiac lymphatics influence local fluid balance, inflammatory resolution, and long‐term graft outcome.

Overall, metabolic and cardiovascular lymphatic dysfunction should be interpreted as coupled disruption of drainage, lipid handling, inflammatory resolution, and tissue repair rather than as an isolated structural defect.

### 4.5 Meningeal Lymphatics in CNS Clearance and Immune Communication

Meningeal LECs form a site‐specialized interface that couples CNS‐derived efflux with peripheral immune communication, rather than serving only as a drainage outlet [94, 96, 358, 359]. The key issue is how meningeal LEC states shape the balance between cerebrospinal fluid (CSF) and waste clearance, antigen delivery, and immune‐cell trafficking across disease contexts. Louveau et al. showed that meningeal lymphatic vessels drain CSF components and support CCR7‐dependent trafficking of immune cells to draining lymph nodes, while meningeal LECs display a distinct transcriptional profile, indicating that this system is specialized for more than fluid outflow alone [94].

This coupling does not produce a uniform biological consequence. The same meningeal lymphatic route can either amplify pathogenic immune priming or support protective immune surveillance, depending on disease context. This contrast is clearest when autoimmunity is compared with brain tumors. In experimental autoimmune encephalomyelitis, impairment of meningeal lymphatic function reduces transport of brain‐derived antigens to deep cervical lymph nodes, attenuates brain‐reactive T‐cell responses, and alleviates disease severity, indicating that this axis can support pathogenic immune priming in autoimmunity [94]. Conversely, VEGFC‐driven enhancement or expansion of meningeal lymphatics in brain tumor or radiotherapy settings promotes antigen delivery to deep cervical lymph nodes, CD8^+^ T‐cell activation, tumor infiltration, and responsiveness to immune checkpoint therapy [358, 360]. Thus, meningeal lymphatics can aggravate autoimmune priming while supporting antitumor immune surveillance in brain tumors.

After tissue injury, the functional priority shifts toward fluid clearance and resolution of inflammatory burden. In stroke, VEGFC‐based enhancement of meningeal lymphatics improves cerebral fluid drainage, modulates neuroinflammation, facilitates cerebrospinal and interstitial fluid outflow, and improves recovery, although timing and the relationship with cerebral edema remain incompletely resolved [361, 362]. A similar clearance‐focused pattern is seen in sepsis‐associated encephalopathy and traumatic brain injury, in which impaired meningeal lymphatic drainage contributes to neuroinflammation, cognitive dysfunction, or edema‐related injury, whereas enhancing this pathway is associated with functional improvement [363, 364, 365, 366]. In these injury settings, restoration of meningeal lymphatic drainage appears most relevant when pathology is dominated by fluid retention, defective waste clearance, and unresolved inflammation.

By comparison, in neurodegenerative disease the meningeal lymphatic system is better viewed as an expanding mechanistic frontier than as a therapeutic axis already supported by definitive causal validation. Although increasing attention has focused on meningeal lymphatic clearance of Alzheimer disease‐related proteins such as Aβ and tau, this area still points more to an emerging regulatory mechanism and possible therapeutic window than to a pathway with established translational support [93, 367, 368, 369].

Overall, meningeal lymphatics should be understood as a context‐dependent LEC interface linking CNS efflux to peripheral immunity. Their functional meaning depends on the dominant pathological demand: enhancing clearance of excess fluid, inflammatory mediators, and CNS‐derived waste may be beneficial in injury or selected neurodegenerative contexts, whereas limiting CNS antigen delivery may be more relevant when peripheral immune priming drives pathology.

## 5 Strategies Targeting LECs in Disease

**FIGURE 4:**
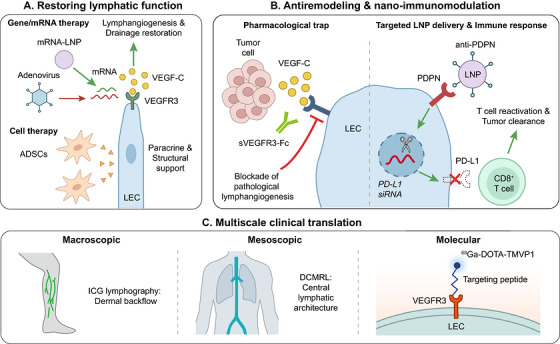
Precision lymphatic medicine: targeted interventions, nanomedicine, and molecular imaging. (A) Restorative strategies include gene‐ or mRNA‐based delivery of VEGFC and cell‐based approaches such as adipose‐derived stromal cell therapy to promote lymphatic repair, improve drainage, and restore transport competence. (B) Restraining and immunomodulatory strategies include pharmacological trapping of VEGFC with sVEGFR3‐Fc to limit pathological sprouting, as well as targeted LNP delivery of PD‐L1 siRNA to LECs to enhance CD8^+^ T‐cell reactivation and tumor clearance. (C) Imaging‐linked translation spans multiple spatial and biological scales, from ICG lymphography for dermal backflow, to DCMRL for central lymphatic architecture, and to molecular PET imaging with VEGFR3‐targeted probes such as ^68^Ga–DOTA–TMVP1. ADSCs, adipose‐derived stromal cells; DCMRL, dynamic contrast‐enhanced magnetic resonance lymphangiography; ICG, indocyanine green; LEC, lymphatic endothelial cell; LNP, lipid nanoparticle; PD‐L1, programmed death‐ligand 1; PDPN, podoplanin; PET, positron emission tomography; siRNA, small interfering RNA; sVEGFR3‐Fc, soluble vascular endothelial growth factor receptor 3‐Fc fusion protein; VEGFC, vascular endothelial growth factor C; VEGFR3, vascular endothelial growth factor receptor 3.

A state‐centered view of LEC biology changes how lymphatic‐directed therapies should be selected and evaluated. Rather than following a generic pro‐ or antilymphatic logic, interventions should be matched to the dominant mode of dysfunction: restoring lymphatic repair when recovery is inadequate, restraining pathological remodeling when LEC reprogramming becomes disease‐promoting, or redirecting LEC‐mediated immune functions when altered immune communication sustains pathology [7, 19, 97, 98, 106, 179, 304, 315, 350]. Precision delivery systems and imaging‐based functional assessment can support this framework by identifying the relevant LEC state, defining the therapeutic window, and monitoring biological and clinical responses [207, 350, 370, 371] (Figure 4).

### 5.1 Restoring Lymphatic Growth and Function

When lymphatic injury occurs, the therapeutic goal is not simply to replace lost vessels, but to rebuild a functionally connected transport network. Restorative strategies should therefore be evaluated by whether they restore sustained clearance, directional flow, valve and collector function, and integration within injured tissue [207, 372, 373] (Figure 4A). Representative approaches include VEGFC–VEGFR3‐directed lymphangiogenesis, gene and mRNA‐based delivery, cell and tissue‐engineering strategies, small molecules, and physical stimulation, each of which should be assessed according to its ability to re‐establish lymphatic growth and function in a specific disease context.

#### 5.1.1 VEGFC–VEGFR3‐Directed Lymphangiogenesis

VEGFC and its homolog VEGFD remain the central molecular axis for restorative lymphatic intervention because they drive lymphatic growth primarily through VEGFR3/FLT4 [294, 374]. In postdevelopmental disease and repair settings, the therapeutic relevance of this pathway depends on whether VEGFC–VEGFR3 signaling can reactivate reparative LEC programs that mature into a connected, low‐leakage, and functionally competent transport network. Consistent with this principle, disruption of VEGFC–VEGFR3 signaling severely impairs lymphatic development, whereas local delivery of VEGFC protein or related genes in injured or diseased tissues can promote lymphatic growth, improve drainage, and alleviate edema and inflammation associated with lymphatic insufficiency [175, 207, 346, 350, 375, 376] (Figure 4A). The key translational issue is therefore whether VEGFC‐induced remodeling restores durable transport competence rather than producing transient or poorly integrated vascular expansion.

This functional output is strongly shaped by ligand processing and tissue context. Full VEGFC activity requires proteolytic maturation through the CCBE1–ADAMTS3 processing axis, making local processing capacity a determinant of whether VEGFC supplementation translates into effective lymphatic regeneration [38, 176, 221, 377, 378, 379, 380, 381, 382]. Consistent with this view, enhancement of CCBE1‐dependent VEGFC processing contributed to restoration of lymphatic function in secondary lymphedema [176]. Thus, therapeutic optimization should consider ligand abundance, proteolytic activation, receptor balance, dose, delivery route, disease stage, and tissue architecture together. Simply increasing VEGFC exposure may be insufficient, or even counterproductive, if the local environment cannot support vessel maturation and network integration.

Safety evaluation is equally context dependent. Excessive, prolonged, or poorly localized VEGFC activity may drive lymphatic expansion before newly formed vessels acquire stable junctional organization, downstream connectivity, and barrier competence, shifting the outcome from productive drainage recovery toward leakage‐prone or maladaptive remodeling [207, 208, 376, 383, 384]. Because VEGFC can also activate VEGFR2, off‐target blood‐vessel permeability and vascular leakage remain important concerns [208, 376]. At the lymphatic level, incomplete junctional maturation or defective network integration may likewise produce lymphatic leakage or ineffective clearance rather than sustained fluid drainage [84, 85, 86, 111, 384]. Therefore, adverse endpoints should be disease specific: persistent dermal backflow or insufficient drainage recovery may be most informative in lymphedema [106, 187]; edema and inflammatory‐cell retention may indicate failed functional repair in inflamed tissues [257, 258, 261]; expanded lymphatic routes may facilitate tumor dissemination [290, 291, 319]; and plaque neovascularization, intraplaque hemorrhage, necrotic‐core expansion, and fibrous‐cap preservation require particular attention in atherosclerotic plaques [342, 343, 344].

VEGFC156S illustrates both the promise and the limitation of receptor‐selective lymphangiogenic therapy. This VEGFR3‐preferring ligand was developed to reduce part of the leakage‐related risk, and in mouse subcutaneous and limb lymphedema models, it induced lymphangiogenesis while reducing vascular leakage and related adverse effects [376]. However, receptor selectivity alone does not guarantee therapeutic superiority. In a porcine model of secondary lymphedema, wild‐type VEGFC more effectively promoted lymphangiogenesis and preserved transplanted lymph node structure than VEGFC156S without causing unacceptable vascular toxicity [385]. Accordingly, the choice among wild‐type VEGFC, VEGFC156S, VEGFD, and related ligands should be guided by an integrated assessment of lymphangiogenic efficacy, vascular safety, lymphatic leakage, delivery route, therapeutic window, disease stage, and organ‐specific lymphatic architecture [207, 361, 383].

#### 5.1.2 Gene Therapy and mRNA‐Based Lymphangiogenic Strategies

If protein supplementation mainly addresses how to trigger lymphangiogenic signaling, gene and nucleic‐acid delivery address a related but distinct therapeutic question: how to sustain VEGFC activity within a defined regenerative window without losing spatial and temporal control. Early VEGFC gene transfer in a rat model of secondary lymphedema induced new lymphatic vessel formation, reduced limb swelling, and improved lymphatic drainage [386]. In a porcine model, local delivery of adenoviral VEGFC or VEGFC156S combined with vascularized lymph node transfer (VLNT) supported reconstruction of a functional lymphatic network, although wild‐type VEGFC produced stronger lymphangiogenesis and better preservation of transferred lymph nodes than VEGFC156S [385]. More recently, nucleoside‐modified VEGFC mRNA‐LNPs induced durable, organ‐specific lymphatic growth and restored lymphatic function in experimental lymphedema [175]. Together, these studies indicate that nucleic‐acid‐based lymphangiogenic therapy is most valuable when it sustains a local reparative LEC program long enough for drainage pathways to reconnect, while avoiding uncontrolled or prolonged growth stimulation.

This concept has already entered early clinical testing, but clinical benefit remains dependent on patient selection, timing, and outcome definition. The clinical‐grade adenoviral VEGFC formulation Lymfactin, delivered in combination with VLNT, showed acceptable short‐term safety and tolerability in Phase I [237]. Longer follow‐up suggested sustained tolerability and functional improvement in some patients, including reductions in limb volume [238], and the final Phase I report further showed reduced compression‐free swelling and improved quality of life over extended follow‐up [239]. However, a randomized Phase II trial did not demonstrate between‐group differences in the primary efficacy endpoints at 12 months, despite quantitatively favorable changes in skin interstitial fluid in the Lymfactin arm [240]. These mixed clinical results suggest that successful translation will require better alignment among disease stage, residual lymphatic architecture, surgical reconstruction, gene‐delivery kinetics, and functional endpoints, rather than simply stronger induction of lymphangiogenesis.

mRNA‐LNP platforms further refine this therapeutic logic by enabling transient, nonintegrating, and locally tunable VEGFC expression. In mouse models, a single low‐dose subcutaneous or local injection of VEGFC mRNA‐LNP induced transient local VEGFC expression, efficient organ‐specific lymphangiogenesis, restored lymphatic drainage, and reversed experimental lymphedema while avoiding the risk of vector‐mediated genomic integration [175]. In myocardial infarction, transient local VEGFC expression delivered by mRNA‐LNP promoted cardiac lymphangiogenesis, attenuated inflammatory and fibrosis‐associated signaling, reduced fibrosis, and improved cardiac function [350]. LEC‐targeted VEGFC mRNA‐LNP delivery further improved lymphatic function after injury and was proposed as a strategy for targeted, transient lymphangiogenic therapy [207]. Compared with permanent or broadly distributed gene expression, this platform is conceptually attractive for conditions in which lymphatic repair is needed within a limited therapeutic window and must be matched to a specific tissue compartment.

Combination approaches may be particularly important when lymphatic failure reflects not only deficient VEGFC signaling, but also disrupted tissue architecture, fibrosis, inflammation, or loss of local regenerative support. VEGFC gene delivery combined with VLNT generated more abundant and functionally favorable lymphatic networks around transplanted lymph nodes in large‐animal studies, and early clinical studies suggest that this combination is feasible, well tolerated, and associated with signals of functional benefit [238, 239, 240, 385]. VEGFC–VEGFR3‐related signals may also be combined with biodegradable collagen or fibrin scaffolds carrying LECs or stem cells to construct engineered lymph nodes or lymphatic conduits that more closely reproduce physiological drainage pathways in severe lymphatic hypoplasia [372, 387, 388]. Other lymphotrophic pathways, including hepatocyte growth factor, adrenomedullin, and fibroblast growth factor 2, can promote lymphatic endothelial proliferation, migration, branching, or edema reduction in preclinical settings [389, 390, 391, 392]. Thus, future restorative strategies should move from single‐signal lymphangiogenesis toward context‐matched regenerative combinations that rebuild both the lymphatic network and its supportive microenvironment [372, 393].

#### 5.1.3 Cell‐Based and Tissue‐Engineering Approaches

Cell‐based and tissue‐engineering approaches represent a restorative strategy that aims to rebuild the lymphatic repair niche rather than deliver a single lymphangiogenic signal. Living cellular populations can provide sustained paracrine support, respond to the local environment, and participate in tissue remodeling [394]. This logic is especially relevant when lymphatic failure reflects not only insufficient vessel growth, but also fibrosis, niche disruption, and incomplete regenerative support. In preclinical models of secondary lymphedema, mesenchymal cells, particularly adipose‐derived stem cells (ADSCs), promoted lymphatic regeneration, reduced fibrosis, and partially restored drainage. ADSC transplantation increased LYVE1^+^ lymphatic vessels and endothelial proliferation, reduced radiation‐induced skin fibrosis, enhanced functional lymphatic branch formation through intussusceptive lymphangiogenesis, and ultimately reduced limb swelling [395]. In another mouse study, ADSC transplantation promoted lymphangiogenesis and reduced edema volume, at least partly through paracrine lymphangiogenic support and increased VEGFC–VEGFR3‐related responses [396]. More complex cellular systems may further improve repair: combined transplantation of mesenchymal stem cells (MSCs) and lymphatic endothelial progenitor cells enhanced efficacy in murine hindlimb lymphedema, whereas bioengineered lymphatic tissues containing LECs and MSCs restored lymph flow and reduced edema in mice [372, 394].

Clinical translation remains promising but still preliminary. A case report of cell‐assisted lipotransfer, in which autologous adipose‐derived stromal/regenerative cells were coinjected with fat grafts into axillary scar tissue, described mild reduction in limb volume and symptomatic improvement without serious adverse events [397]. A prospective Phase I feasibility and safety study in 10 patients with breast cancer‐related lymphedema similarly delivered autologous adipose‐derived regenerative cells with fat grafts into the axillary region; the procedure was generally well tolerated, and some patients reported symptomatic improvement together with reduced need for conservative therapy. Yet the mean reduction in limb volume was small and did not reach statistical significance, and subsequent 1‐year and 4‐year follow‐up studies likewise failed to show significant volume reduction despite persistent patient‐reported benefit [398, 399, 400, 401]. Thus, current clinical evidence supports feasibility and safety more strongly than reproducible efficacy.

The key translational question is therefore under which biological and practical conditions cell‐based repair can produce durable functional benefit. These conditions include cell source, the choice between ex vivo expansion and immediate isolation, dose, route of administration, persistence of the delivered cells, interaction with host LECs, and long‐term oncological safety [398, 399, 401, 402]. Future studies should combine larger randomized trials and longer follow‐up with mechanistic endpoints that distinguish paracrine lymphangiogenic or antifibrotic support, cooperation with host lymphatic cells, and partial acquisition of lymphatic endothelial characteristics [394, 395, 396, 403, 404]. Only with these issues better resolved will it be possible to define an effective therapeutic window while limiting risks such as ectopic vascular growth, aberrant lymphatic remodeling, or oncological recurrence.

#### 5.1.4 Small‐Molecule and Physical Approaches

Small‐molecule and physical interventions are best framed as indirect, repair‐supporting approaches that modulate the inflammatory, fibrotic, metabolic, or mechanical milieu in which LEC repair occurs, rather than as direct substitutes for lymphangiogenic growth programs. Pharmacological evidence supports this permissive‐repair model. Leukotriene B4 blockade or 5‐lipoxygenase inhibition can alleviate lymphatic structural injury and improve drainage in chronic lymphedema models, whereas β‐hydroxybutyrate enhances OXCT1‐dependent mitochondrial oxidative capacity in LECs and supports lymphatic reconstruction after myocardial infarction and acquired lymphatic injury [405, 406]. Clinical and experimental findings further suggest that systemic metabolic state can influence lymphatic repair, because ketogenic intervention may improve lymphatic function in some patients with secondary lymphedema, while saturated fatty acids can injure LECs and worsen experimental disease [407, 408]. Together, these findings indicate that inflammatory and metabolic cues can either enable or restrain reparative LEC function.

Drug repurposing and physical stimulation extend this repair‐supporting logic but remain exploratory. Cilostazol, 9‐cis retinoic acid, hyaluronidase, and ketoprofen have improved lymphatic structure or function in experimental models or small exploratory studies, mainly by promoting lymphangiogenesis or reducing inflammation and fibrosis [236, 409, 410, 411, 412, 413, 414, 415]. Physical stimulation provides another repair‐supporting route, as exercise and low‐energy extracorporeal shock wave therapy have been reported to improve lymphatic drainage, vessel remodeling, edema, or fibrosis in preclinical models and small clinical studies [416, 417, 418, 419]. However, because long‐term efficacy, mechanism specificity, stimulation parameters, tissue tolerance, and safety remain insufficiently defined, these approaches should currently be viewed as adjunctive strategies that create a repair‐permissive environment rather than as established LEC‐directed regenerative therapies.

Overall, restorative prolymphangiogenic therapy should be guided not simply by whether lymphatic growth can be induced, but by when, where, and to what extent repair should be promoted. Because lymphangiogenesis may restore drainage in regenerative settings but facilitate dissemination in tumors, these strategies should be advanced only when durable functional repair is expected to outweigh metastatic or maladaptive risk [290, 291, 292, 295, 318, 420].

### 5.2 Targeting Pathological Lymphatic Remodeling

Pathological lymphatic remodeling becomes a therapeutic target when remodeled LEC networks acquire disease‐promoting trafficking, dissemination, or immune‐regulatory functions, rather than simply because lymphatic vessel density increases. Once remodeling becomes permissive for dissemination, immune evasion, or excessive immune activation, the therapeutic task shifts from repair to restraint [97, 194, 421, 422, 423, 424]. Antilymphangiogenic intervention should therefore be viewed not as the simple inverse of prolymphangiogenic therapy, but as a strategy to disrupt disease‐conditioned LEC states after lymphatic remodeling has shifted from adaptation to pathology. Tumor biology provides some of the clearest support for this framework. In cervical cancer, tumor‐associated macrophage expression of VEGFC and VEGFD correlated with increased peritumoral lymphatic microvessel density and higher risk of lymph node metastasis [425]. More broadly, tumor‐associated lymphangiogenesis reflects sustained crosstalk among tumor cells, myeloid populations, stromal components, and LECs, with remodeled lymphatic vessels reinforcing immune suppression and metastatic spread [97, 194, 421, 422].

In cancer, the objective is therefore not merely to suppress LEC proliferation, but to dismantle a remodeled lymphatic niche that supports tumor‐cell intravasation, nodal spread, and immune suppression. Within this framework, the VEGFC–VEGFR3 axis remains one of the best‐defined intervention points. Lin et al. used an adeno‐associated virus to express the soluble VEGFR3 decoy receptor sVEGFR3‐Fc and observed marked reductions in tumor‐associated lymphangiogenesis and lymph node metastasis [196]. This indicates that VEGFC–VEGFR3 blockade can weaken the lymphatic route exploited for metastatic dissemination, even when effects on primary tumor size are limited [420]. More recent work further suggests that antilymphangiogenic therapy may be most useful in combination settings, where it can limit metastatic spread, reshape the tumor microenvironment, and improve responsiveness to anticancer treatment [420, 426].

Selective functional inhibition may, in some contexts, be preferable to maximal pathway blockade. NRP2, a VEGFC coreceptor, offers such an alternative point of intervention. Anti‐NRP2 antibodies inhibited VEGFC‐induced lymphatic endothelial migration, reduced functional tumor‐associated lymphangiogenesis, and decreased both sentinel lymph node and distant metastases, while exerting relatively limited effects on normal adult lymphatic vessels [427]. These findings suggest that disease‐promoting lymphatic remodeling can sometimes be restrained by limiting migration, network connectivity, or functional remodeling, rather than by completely suppressing lymphangiogenesis. Future antilymphangiogenic strategies may therefore need to prioritize selective disruption of pathological lymphatic function over broad inhibition of all lymphatic growth.

The same principle extends beyond cancer, especially when remodeled lymphatic networks sustain pathological antigen export, APC trafficking, or downstream immune priming. Blocking VEGFR3 signaling reduced APC migration to draining lymph nodes and alleviated delayed‐type hypersensitivity as well as corneal transplant rejection [428]. Subsequent studies further showed that VEGFC is upregulated during corneal allograft rejection, and that VEGFC neutralization suppresses both vascular and lymphatic responses while inhibiting APC migration and maturation, thereby improving graft survival [429]. In these settings, antilymphangiogenic intervention acts not only on vessel abundance, but also on antigen drainage and the immune consequences that follow. However, the same intervention may restrain pathological priming in one context while impairing beneficial drainage, tissue repair, or immune resolution in another [374, 430].

Clinical translation remains the major bottleneck for this strategy. In a Phase I study, the anti‐VEGFR3 monoclonal antibody LY3022856 was generally tolerated in patients with advanced solid tumors and in an expanded colorectal cancer cohort, but no clear objective responses were observed and overall antitumor activity remained limited [431]. These findings suggest that the value of antilymphangiogenic therapy is unlikely to be captured by short‐term primary tumor shrinkage alone. More plausibly, therapeutic benefit will depend on delaying metastatic progression, altering tumor or immune microenvironmental states, or increasing sensitivity to combination therapy [426, 432]. Advancing this field will therefore require sharper patient stratification, better timing of intervention, rational combinations, and endpoints that measure metastatic behavior, lymphatic remodeling, immune‐state changes, and preserved tissue drainage rather than primary tumor size alone.

### 5.3 Therapeutic Immunomodulation via LECs

LECs should no longer be viewed mainly as structural linings of lymphatic vessels, because their therapeutic relevance increasingly lies in their ability to shape antigen handling, T‐cell fate, and peripheral tolerance through antigen presentation, chemokine production, and coinhibitory signaling [17, 98, 433]. For therapeutic purposes, however, this immune function must be interpreted by lymphatic bed, LEC state, and immune module rather than as a generic property of all lymphatic vessels. Single‐cell transcriptomic and lineage‐tracing studies have shown that lymph node LECs comprise spatially and functionally distinct subsets, including subcapsular, cortical, and medullary populations specialized for antigen capture, antigen archiving, or chemokine‐network organization [5, 14]. Related state specialization is also seen in the skin, intestine, meninges, and heart [13, 57, 95, 181, 434]. Thus, LEC‐directed immunomodulation should be understood as context‐specific immune redirection rather than broad activation or inhibition of lymphatic vessels.

Within lymph nodes, the best‐defined immunoregulatory program is tolerogenic. Resident LECs can transcribe and present tissue‐restricted self‐antigens to CD8^+^ T cells through MHC Class I, yet provide little conventional costimulation while expressing high levels of PD‐L1. Under these conditions, antigen presentation drives deletion or functional inactivation rather than effector differentiation of autoreactive T cells [16, 17]. LECs can also retain antigens derived from pathogens or vaccines for prolonged periods, creating antigen archives that later reshape memory T‐cell responses upon renewed antigen encounter [5, 76, 77]. Consistently, systemic interruption of the PD‐1/PD‐L1 pathway disrupts this tolerogenic program and allows CD8^+^ T cells recognizing LEC‐expressed self‐antigens to acquire effector activity and induce autoimmune tissue injury [16]. These findings position lymph node LECs as active regulators of whether antigen exposure is routed toward tolerance, memory recall, or effector immunity.

Outside lymph nodes, the therapeutic meaning of LEC immune function can shift from tolerance control to immune access, drainage, or antitumor priming. The meningeal lymphatic system provides a particularly instructive example: enhancing meningeal drainage can improve immune surveillance in brain tumors, increase responsiveness to immune checkpoint therapy, and may also augment radiotherapy‐associated antitumor immunity [358, 360]. This context does not contradict the tolerogenic role of lymph node LECs; rather, it shows that different lymphatic beds control different immune bottlenecks.

Tumors represent a different configuration, in which lymphatic remodeling often shifts toward immune suppression and dissemination. Tumor‐derived factors such as VEGFC promote local lymphatic expansion, while tumor‐associated LECs acquire a more immunosuppressive phenotype that weakens antitumor immunity [97, 99, 194, 422]. In part through local IFN‐γ signaling, these LECs upregulate PD‐L1 and other inhibitory molecules, suppress activated T‐cell proliferation and effector function, and reshape transendothelial migration of regulatory and effector T‐cell subsets [98, 99, 157]. More direct evidence comes from LEC‐specific PD‐L1 knockout models, in which loss of PD‐L1 on lymphatic endothelium enhances the expansion of tumor‐specific CD8^+^ T cells in tumor‐draining lymph nodes, reduces tumor burden, and improves adoptive T‐cell therapy, with the dominant effect linked to reduced apoptosis of tumor‐specific memory T cells [98]. Thus, a tumor‐conditioned LEC state can actively enforce immune escape rather than merely accompany tumor progression.

Yet tumor‐associated LECs should not be reduced to a uniformly suppressive role. In melanoma and related models, VEGFC‐driven lymphatic expansion has also been linked to greater responsiveness to PD‐1 blockade [435, 436]. One explanation is that a functionally intact lymphatic network improves DC and lymphocyte trafficking into and through the tumor. Another is that LEC‐derived chemokines such as CCL21 promote lymphocyte recruitment, thereby increasing both the abundance and activation state of tumor‐infiltrating lymphocytes under checkpoint blockade [435]. Histological and clinical correlative studies likewise suggest that, in some solid tumors, higher lymphatic vessel density or VEGFC‐associated lymphatic signatures are associated with stronger immune‐cell infiltration and, in melanoma, better responsiveness to checkpoint blockade [435, 436, 437]. The immunological consequence of tumor lymphatics therefore depends on whether lymphatic remodeling predominantly supports antigen drainage and immune‐cell entry or enforces local checkpoint‐mediated suppression.

These context‐dependent states create two therapeutic directions. In tumors, the goal is to relieve LEC‐mediated suppression while preserving, or even strengthening, lymphatic functions that support antigen drainage and immune‐cell entry. In principle, targeting PD‐L1 on LECs or its downstream signaling network could complement existing PD‐1/PD‐L1 therapies [98]. Because PD‐L1 also regulates T‐cell traversal across lymphatic endothelium [157], local or LEC‐selective blockade might additionally reshape lymphatic migratory routes in ways that improve immune‐cell positioning. In autoimmunity or transplantation, the therapeutic logic is reversed: reinforcing tolerogenic LEC programs may help restrain pathological immune activation, although this concept remains supported mainly by theoretical reasoning and early animal work rather than systematic preclinical validation [16, 17, 438]. The relevant LEC subsets, tissue sites, and regulatory mechanisms are also unlikely to be identical across RA, IBD, and transplantation [126, 256, 438, 439].

Thus, LEC‐directed immunomodulation should be advanced as state‐aware immune modulation rather than broad enhancement or inhibition of lymphatic activity. The main translational barrier is specificity: generalized checkpoint manipulation carries systemic autoimmune risk, whereas nanoparticles, viral vectors, or other delivery systems directed toward LEC‐associated markers may provide a route toward more localized modulation of lymphatic endothelial checkpoint pathways [207, 440]. This logic also favors combination and stratification strategies rather than standalone intervention. In tumors, VEGFC‐driven lymphatic support may need to be coupled with LEC‐directed checkpoint modulation [98, 435], while intratumoral lymphatic density and VEGFC‐associated signatures may help identify immune‐inflamed states more likely to respond to immunotherapy [435, 437]. Ultimately, progress will depend on state‐aware delivery, patient stratification, and response monitoring so that LEC‐directed immunomodulation can be applied in a controllable and verifiable manner across cancer, infection, inflammatory disease, and transplantation.

### 5.4 Nanomedicine for LEC‐Targeted Delivery: Promise and Constraints

Nanomedicine is best positioned in LEC‐directed therapy not as an independent therapeutic category, but as an enabling platform for executing state‐selective intervention. The central limitation of many lymphatic strategies is not the absence of candidate payloads, but the difficulty of delivering them with enough spatial, cellular, and temporal precision to avoid broad manipulation of lymphatic tissue. This is particularly important because reparative, inflammatory, immunoregulatory, and disease‐promoting LEC states may coexist within the same pathological setting. The key translational question is therefore whether a nanoplatform can engage the relevant LEC population at the appropriate disease stage and reshape defined functional modules, including proliferation, migration, barrier behavior, immune crosstalk, or reparative responses, rather than simply whether it can reach lymphatic‐associated tissue (Figure 4B).

Early nanocarrier studies mainly established delivery feasibility rather than true state‐ or cell‐selective intervention [441, 442, 443]. A PEI–alginate nanocomposite carrying VEGFR3 siRNA reduced VEGFR3 expression, inhibited differentiation of endothelial progenitor cells toward a lymphatic endothelial‐like phenotype, and suppressed lymphatic‐like migration and tubule formation in an EPC differentiation model [443]. Although this work provided proof of concept for nanocarrier‐mediated pathway interference, it did not establish selective targeting of mature LECs within an authentic pathological microenvironment. More recent antibody‐modified or compositionally optimized LNPs have begun to address this limitation by enabling siRNA or mRNA delivery to LECs in vivo after local administration [440, 444].

A more meaningful conceptual advance came with surface‐guided and formulation‐guided LNP design, which shifted the field from coarse lymphatic enrichment toward marker‐informed interaction with target endothelial populations. A CLIP‐based strategy for rapidly conjugating antibodies onto the LNP surface used anti‐PDPN‐modified LNPs as a representative example and demonstrated siRNA delivery to LECs both in vitro and in vivo [440]. This approach is important because it links surface‐marker choice, uptake route, and therapeutic cargo to defined LEC populations. In parallel, compositionally optimized LNPs achieved ApoE‐mediated siRNA delivery to LECs in vivo, and LNP library screening identified formulations with stronger LEC tropism for therapeutic VEGFC mRNA delivery after lymphatic injury [207, 444]. These studies move lymphatic nanomedicine closer to a model in which delivery platforms are designed to distinguish functionally relevant LEC populations rather than merely accumulate near lymphatic tissue.

This design logic becomes especially important when nanomedicine is used to actively reprogram LEC behavior rather than only suppress lymphatic signaling. In a mouse model of lymphatic injury, LEC‐targeted delivery of VEGFC mRNA promoted proliferation of LECs within the injured region and improved lymphatic function after a single administration, with effects persisting for up to 2 weeks after surgery [175, 207]. This extends earlier evidence that VEGFC mRNA‐LNPs can induce organ‐specific lymphatic growth and reverse experimental lymphedema, and that VEGFC mRNA nanodelivery can promote reparative lymphangiogenesis after myocardial infarction [175, 350]. The therapeutic meaning of this strategy, however, depends on disease context. VEGFC‐driven repair may be desirable when lymphatic insufficiency limits drainage or tissue recovery, but the same growth‐promoting axis could be problematic in settings where lymphatic expansion, inflammatory remodeling, or tumor‐associated lymphangiogenesis supports disease progression [290, 291, 319]. Thus, reparative mRNA nanotherapy should be interpreted as a stage‐ and context‐dependent strategy rather than a broadly prolymphangiogenic intervention.

Despite these advances, the field remains far from clinical maturity. Most supporting evidence still comes from animal models using local administration, whereas human tissues impose greater complexity in anatomical structure, drainage kinetics, lesion accessibility, and tolerance to repeated exposure. Many platforms described as LEC‐targeted still achieve relative enrichment rather than true cellular specificity [175, 207, 350, 440, 444]. This leaves unresolved concerns about off‐target uptake by non‐LEC populations, effective dose windows, repeated dosing, immune tolerance, and the durability of LEC state changes after treatment. The next phase of LEC‐directed nanomedicine should therefore move beyond improved delivery chemistry alone and integrate nanoparticle design with single‐cell state mapping, imaging‐based response monitoring, and patient stratification. Ultimately, success will depend on whether delivery can be matched to the right LEC subset, organ context, disease stage, and functional state with sufficient precision, safety, and verifiable biological effect.

### 5.5 Imaging as a Bridge to Clinical Translation

For LEC‐directed therapy, the translational challenge does not end with demonstrating that a pathway is mechanistically relevant. It also requires determining which patients display a lymphatic dysfunction phenotype that is clinically measurable, mechanistically linked to disease progression, and potentially reversible after intervention [370, 445, 446]. Imaging therefore provides more than diagnostic support; it supplies the clinical framework that connects mechanistic stratification, treatment selection, and objective response assessment. This role is clearest in lymphedema and is increasingly relevant in other disease settings where drainage failure, reflux, central lymphatic overload, or lymphatic molecular activation may define distinct therapeutic windows [370, 445, 446, 447, 448, 449] (Figure 4C).

At present, the most clinically mature tools remain functional and dynamic imaging modalities, including indocyanine green (ICG) lymphangiography, lymphoscintigraphy, and MR lymphangiography [371, 450, 451, 452]. Near‐infrared ICG lymphangiography is now among the most widely used methods for superficial lymphatic imaging in clinical practice. Existing ICG‐based classification and staging systems correlate dermal backflow patterns with the severity of lower‐extremity lymphedema and provide an actionable framework for surgical decision‐making, longitudinal follow‐up, and individualized conservative management [445, 449, 452, 453]. When disease involves deep or central lymphatic pathways, dynamic contrast‐enhanced MR lymphangiography (DCMRL) extends this assessment by defining central lymphatic routes, reflux patterns, and complex drainage abnormalities that are not readily captured by superficial imaging alone [371, 450]. Thus, ICG and DCMRL should be viewed not as competing modalities, but as complementary readouts of different levels of lymphatic dysfunction, from superficial transport failure to central routing abnormalities.

Nuclear medicine approaches further extend this framework by enabling more quantitative and whole‐system assessment. For example, ^6^ ^8^Ga‐NOTA‐Evans Blue positron emission tomography/computed tomography (PET/CT) may provide more comprehensive information than conventional lymphatic imaging in lymphangioleiomyomatosis, particularly for pulmonary and extrapulmonary lymphatic abnormalities [454]. Together, ICG, DCMRL, and PET/CT mark a shift from anatomical depiction toward function‐oriented assessment of transport burden, reflux, and system‐level abnormality. Consistent with this view, recent DCMRL studies have shown that imaging can help define underlying central lymphatic abnormalities and predict both the feasibility of lymphatic intervention and clinical outcome [371].

Building on functional imaging, molecular PET tracers directed at lymphatic targets may move the field closer to molecularly informed stratification. The VEGFR3‐binding TMVP1 peptide tracer ^6^ ^8^Ga‐DOTA‐TMVP1, based on the pentapeptide sequence LARGR, has been evaluated in animal models and small clinical cohorts, with tracer accumulation above background observed in most identified lesions from patients with recurrent gynecological tumors [455]. This represents one of the earlier efforts to bring VEGFR3‐related molecular activity into clinical PET imaging. Its value lies not simply in identifying additional lesions, but in making lymphatic‐associated molecular activity visible in vivo, thereby creating a potential bridge between LEC biology and patient selection. More recent work using the dimeric VEGFR3‐targeted tracer [(^6^ ^8^Ga)Ga‐NOTA‐(TMVP1)_2_] further indicated that VEGFR3 PET may support molecular stratification in ovarian cancer, with 26 of 44 lesions showing positive uptake and preliminary correspondence between tracer signal and VEGFR3 expression in sampled tissues [456]. If prospectively validated, this direction may support companion‐diagnostic development for LEC‐directed therapies, although its ability to guide treatment selection and predict therapeutic response remains to be established.

Even so, imaging remains a major bottleneck in clinical translation. Many current studies still rely heavily on histological readouts, such as lymphatic vessel density or immunostained area, whereas clinical deployment requires robust, reproducible, and cross‐center comparable measures of lymphatic function and treatment response. These include graded reflux patterns, abnormal central lymphatic load, tracer uptake parameters, and longitudinal changes after intervention. Existing platforms, including ICG, DCMRL, and PET/CT, have already laid the groundwork for such an evaluation system, but standardized acquisition protocols, harmonized interpretation criteria, and validated links between imaging readouts and clinical benefit still need to be established more rigorously [370, 371, 445, 449, 457]. Thus, the main translational obstacle is not only the limited availability of effective interventions, but also the lack of an imaging framework that can identify treatable lymphatic dysfunction, guide patient selection, monitor biological response, and compare outcomes across studies. In this sense, imaging is not downstream of LEC‐targeted therapy; it is part of the translational logic that determines whether an intervention is interpretable, adjustable, and clinically verifiable (Table 3).

**TABLE 3:** Therapeutic and translational strategies targeting LEC dysfunction.

Therapeutic logic	Disease context	Representative strategies	Evidence	Key limitations	References
Restore lymphatic repair	Acquired lymphatic insufficiency; impaired tissue repair	VEGFC, VEGFC156S, adenoviral VEGFC, Lymfactin + VLNT, VEGFC mRNA‐LNP	Preclinical; Phase I/II for Lymfactin + VLNT; emerging mRNA‐LNP data	Dose, timing, leakage, durability, endpoint sensitivity	[175, 176, 207, 237, 238, 239, 240, 330, 350, 375, 383, 385, 458, 459, 460]
Support repair niches	Lymphedema; chronic inflammatory disease; fibrotic disease	HGF, adrenomedullin, bFGF, LTB4 blockade, metabolic or physical approaches	Mainly preclinical; selected exploratory clinical data	Low LEC specificity, stage dependence, uncertain durability	[389, 390, 391, 392, 405, 406, 407, 408, 416, 417, 461, 462]
Reconstruct lymphatic architecture	Postsurgical lymphedema; primary lymphatic disease; organ transplantation	ADSCs, MSC–LEC/progenitor combinations, conduits, engineered lymphatic tissues, artificial lymph nodes	Mainly preclinical; early feasibility for selected cell‐based strategies	Integration, long‐term function, fibrosis, oncological safety	[372, 387, 388, 394, 395, 396, 397, 398, 399, 401, 402, 403, 404, 463, 464, 465, 466, 467]
Restrain pathological remodeling	Cancer; lymphatic metastasis	sVEGFR3‐Fc, anti‐VEGFR3, anti‐NRP2, VEGFC neutralization	Preclinical and early translational	Impaired drainage, repair, and immune surveillance	[196, 267, 420, 426, 427, 468]
Reprogram immune functions	Cancer; transplantation; inflammatory disease	PD‐L1–PD‐1 targeting, antigen‐presentation modulation, stromal–lymphatic checkpoint modulation, PD‐L1 siRNA LNP, anti‐podoplanin LNP	Mechanistic and preclinical	Off‐target delivery, tolerance disruption, formulation scalability	[8, 16, 97, 98, 157, 169, 192, 437, 440, 444]
Guide precision translation	Lymphedema; central lymphatic disorders; cancer	ICG lymphography, DCMRL, PET/CT, VEGFR3‐targeted PET tracers	Clinical use for ICG/DCMRL; emerging PET imaging	Standardization, quantification, imaging–outcome validation	[371, 452, 453, 454, 455, 456, 469]

*Abbreviations*: ADSC, adipose‐derived stromal cell; bFGF, basic fibroblast growth factor; DCMRL, dynamic contrast‐enhanced magnetic resonance lymphangiography; HGF, hepatocyte growth factor; ICG, indocyanine green; LEC, lymphatic endothelial cell; LNP, lipid nanoparticle; LTB4, leukotriene B4; MSC, mesenchymal stem/stromal cell; NRP2, neuropilin 2; PD‐1, programmed cell death protein 1; PD‐L1, programmed death‐ligand 1; PET/CT, positron emission tomography/computed tomography; siRNA, small interfering RNA; sVEGFR3‐Fc, soluble VEGFR3‐Fc fusion protein; VEGFC, vascular endothelial growth factor C; VEGFR3, vascular endothelial growth factor receptor 3; VLNT, vascularized lymph node transfer.

## 6 Conclusions, Challenges, Limitations, and Future Perspectives

### 6.1 Conceptual Synthesis and Translational Implications

This review proposes a state‐centered framework for understanding LEC biology. Rather than being defined solely by vessel structure [13, 14, 15, 92, 424], LEC function emerges from the integration of developmental origin, molecular identity, anatomical niche, and context‐dependent cellular state. Within this framework, specialized LEC programs coordinate fluid drainage, lipid transport, immune‐cell trafficking, antigen handling, peripheral tolerance, and tissue repair [24, 61, 87, 424, 470, 471]. In disease, these same programs can be selectively reconfigured, causing lymphatic remodeling to shift from an adaptive response to a maladaptive driver of inflammation, immune dysregulation, metabolic imbalance, tumor progression, or organ dysfunction [15, 86, 96, 129, 140, 290, 291, 472].

This state‐centered framework helps reconcile seemingly divergent observations across disease settings. Depending on tissue context and disease stage, lymphatic remodeling may either promote drainage recovery, inflammation resolution, tissue repair, and fibrosis control or contribute to tumor dissemination, chronic immune activation, or immune suppression [175, 290, 291, 319, 350, 424, 473]. Consequently, these findings suggest that the biological and therapeutic significance of lymphatic remodeling is determined not simply by structural changes, but by the functional state of LECs and their context‐dependent roles in maintaining tissue homeostasis.

### 6.2 Challenges and Limitations in Defining Functional LEC States

Despite these conceptual and translational advances, three major challenges remain. First, although single‐cell and spatial technologies have greatly expanded the catalogue of LEC subsets and states, most remain defined by transcriptional signatures, anatomical location, or disease association rather than function. Second, much of the current mechanistic framework still derives from animal models, whereas longitudinal analyses of human LEC heterogeneity and disease evolution remain limited. Human lymphatic diseases are substantially more heterogeneous owing to anatomical complexity, comorbidities, treatment history, and limited tissue accessibility, making it difficult to infer causality from cross‐sectional datasets alone. Third, current therapeutic strategies and evaluation criteria have not kept pace with advances in LEC biology. Most interventions still target the lymphatic system broadly rather than selectively modulating defined LEC subsets or functional states, while commonly used structural endpoints, such as vessel density or marker expression, remain poor surrogates for lymphatic function.

### 6.3 Future Perspectives Toward Precision Lymphatic Medicine

Future progress will depend on integrating four complementary dimensions: molecular state, anatomical niche, functional output, and clinical phenotype. Integrating single‐cell and spatial omics with lineage tracing, perturbation studies, functional imaging, and longitudinal patient cohorts will enable causal validation of LEC states and facilitate their translation into clinically actionable biomarkers and therapeutic targets. Rather than nonspecific manipulation of the lymphatic vasculature, future therapies should selectively restore, restrain, or reprogram disease‐relevant LEC states according to disease stage, tissue context, and functional requirements.

Collectively, these advances will transform LEC biology from a descriptive discipline into a mechanism‐based and therapeutically actionable framework, establishing the foundation for precision lymphatic medicine.

## Author Contributions

Conception: XR, SL, and JZ. Manuscript preparation: DT, WL, HL, JZ, SL, and XR. All authors have read and approved the final manuscript.

## Funding

XR was supported by the National Natural Science Foundation of China (82370465 and 82170470) and the Hubei Key R&D project (2023BCB013). The funders had no role in study design or preparation of the manuscript.

## Ethics Statement

The authors have nothing to report.

## Conflicts of Interest

The authors declare no conflicts of interest.

## Data Availability

The authors have nothing to report.
